# Analytical Approaches to the Rapid Characterisation of Marine Glycolipids in Bioproduct Discovery

**DOI:** 10.3390/md23090352

**Published:** 2025-08-30

**Authors:** Sudarshan Dhakal, Tim D. Nalder, Susan N. Marshall, Colin J. Barrow

**Affiliations:** 1 Centre for Sustainable Bioproducts, Deakin University, Geelong 3216, Australia; sudarshan.dhakal@deakin.edu.au (S.D.); tdnalder@gmail.com (T.D.N.); 2 Plant & Food Research Group, New Zealand Institute for Bioeconomy Science Limited, Nelson 7010, New Zealand; susan.marshall@plantandfood.co.nz

**Keywords:** marine glycolipids, glycolipid extraction, glycolipid analysis, TLC, LC-MS/MS, marine cerebrosides, marine gangliosides

## Abstract

Glycolipids are structurally diverse amphiphilic molecules with potential as non-petrochemical-derived bioproducts, including surfactants, emulsifiers, and antioxidants. The different bioactivities associated with this range of glycolipid structures also present opportunities for dietary supplements, cosmetics, and pharmaceuticals. Marine glycolipids are underexplored due to challenges with purification and structural characterisation. Analytical approaches enabling efficient sample purification, isolation, and identification of target glycolipids are crucial to determining the bioactivity and functions of organisms such as shellfish and seaweed. This review summarises advances in analytical methods applicable to marine glycolipids, including extraction and enrichment methods tailored to specific subclasses. Thin-layer chromatography (TLC)-based rapid detection techniques developed for specific subclasses in complex biological samples are discussed, alongside structure identification methods based on liquid chromatography (LC)–electrospray ionisation (ESI)–tandem mass spectrometry (MS/MS). Hydrophilic interaction liquid chromatography (HILIC), reverse-phase liquid chromatography (RPLC), and supercritical fluid chromatography (SFC) coupled with MS detection are reviewed for their application to glycolipids. The application of two-dimensional liquid chromatography (2D-LC) and advanced MS-based approaches that facilitate both the rapid resolution and comprehensive characterisation of molecular species are also reviewed.

## 1. Introduction

Glycolipids are a group of biologically active membrane lipids that are ubiquitous in living organisms. They are characterised by the presence of a glycosidic linkage between a hydrophilic sugar head and a hydrophobic lipid tail. The amphiphilic nature of glycolipids has been harnessed for a range of bioproduct applications, including surfactants, emulsifiers, drug delivery systems, dietary supplements, and cosmetics [1,2,3,4]. Marine glycolipids come from a wide range of organisms and are very structurally diverse, making them targets for the development of bioactive and functional products [5,6]. This diversity is a result of complex and varied biosynthetic pathways, the range of environments where source-organisms are found, growth conditions, and evolutionary hierarchy [7,8,9]. Marine glycolipids can be broadly classified into two groups, glycoglycerolipids (GGLs) and glycosphingolipids (GSLs), based on the nature of lipidic fragment [5,10]. Variations can be observed not only in terms of chain length and unsaturation of the lipidic tail, but also in the complexity of glycan head groups.

**Figure 1:**
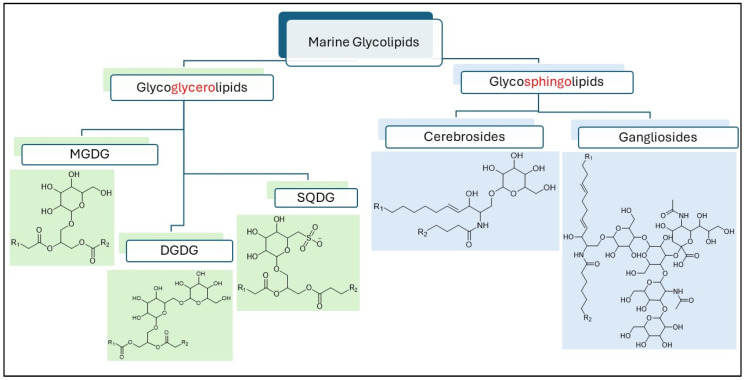
Types of marine glycolipids and their chemical structures. Monogalactosyldiacylglycerol (MGDG), digalactosyldiacylglycerol (DGDG), and sulfoquinovosyldiacylglycerol (SQDG).

GGLs consists of a glyceride base, and are commonly found in phytoplankton, microalgae, and seaweeds [6,11]. They are categorised into three major subclasses, monogalactosyldiacylglycerol (MGDG), digalactosyldiacylglycerol (DGDG), and sulfoquinovosyldiacylglycerol (SQDG), based on the type of sugar head (Figure 1) [12,13]. Modified lyso-forms of GGLs, such as monogalactosylmonoacylglycerol (MGMG), digalactosylmonoacylglycerol (DGMG), sulfoquinovosylmonoacylglycerol (SQMG), and acylated GGLs, are also present in marine algae [14,15,16]. GGLs are reported to possess antimicrobial, anti-inflammatory, and anticancer properties, with numerous molecular structures being identified [13,17,18,19].

GSLs are glycosylated ceramides consisting of a long-chain amino alcohol, often referred as a long-chain base (LCB) or sphingoid base. Cerebrosides and gangliosides (Figure 1) are two types of GSLs present in marine animals such as fish and shellfish [5,10,20]. Glucosylceramides (gluCer) are the most abundant cerebroside subclass present in nature, with other forms such as galactosylceramides (galCer), sulphated hexosylceramides, and cerebrosides with multiple sugar units found less commonly [5,21]. Marine cerebrosides are also known to contain branched sphingoid bases with multiple unsaturation and hydroxy fatty acids, possessing unique bioactivity, such as antitumor [22,23], antiadipogenic [24], and functional properties, including skin barrier improvement [25] and skin hydration effects [26].

Gangliosides are more complex anionic GSLs that are divided into monosialo (GM), disialo (GD), trisialo (GT), and tetrasialo (GQ) subclasses based on the number of sialylated sugar units [27,28,29]. The number of neutral sugars, ‘n’, present in each subclass is indicated as 5-n, following the subclass abbreviation; for example, subclass ‘GM1’ has monosialic acid and four neutral sugars in the glycan head. Additionally, the sialic acid linkage position is indicated by the letters a, b, or c following the subclass abbreviation, for example, GM1a. Marine gangliosides are particularly distinct in terms of both head group modification (acetylation or sulfation) and sphingoid base features (branching and number of unsaturations), making them unique from the mammalian gangliosides [5,20,30,31]. Gangliosides from marine organisms are associated with specific bioactivities of therapeutic relevance, such as neuritogenic activities [30,32].

Biological activity of glycolipid is attributed to the structure of glyco-moieties, their anomeric configuration, and unsaturation in the lipidic chain [22,32,33]. Traditionally, these structures have been investigated using multistep purification protocols involving sequential extraction, column chromatography, multistage derivatisation reactions, and subsequent structural characterisation using chemical and biochemical methods [34,35]. These techniques are not suitable for rapid screening, as they limit assessment of intact structural information associated with specific functional properties. In recent years, liquid chromatography (LC)–electrospray ionisation (ESI)–tandem mass spectrometry (MS/MS) techniques have enabled the rapid identification of intact glycolipid molecular species without the need for derivatisation [7,9,35,36,37]. Lipid class-specific purification techniques are still critical for LC-MS-based analysis to enhance the sensitivity of detection [9,37]. Lower enrichment of glycolipids in total lipid extracts compared to other lipid classes is one of the reasons for difficulty in the detection of glycolipids. As glycolipids are naturally less abundant and exhibit a wider polarity range, associated with their distinct structures, selective extraction can be challenging [10,18]. They are embedded in membranes of cells/tissues, and are bound to other biomolecules such as carbohydrates, proteins, pigments, and phospholipids through H-bonding and ionic interactions [38]. Limited recovery of glycolipids from biphasic extractions is associated with their amphiphilic nature and tendency to form emulsions [39]. Altered phase behaviour can be more pronounced in marine samples due to a higher abundance of complex carbohydrates, for example, in seaweeds [40], or higher concentration of phospholipids, such as in some shellfish [41,42]. Additionally, co-extracted pigments or phospholipids can suppress the ionisation/detection of less abundant glycolipids in ESI-MS [43,44,45]. This has prompted the development of targeted enrichment and sensitive detection methods specific to glycolipid subclasses, such as SQDG [46,47]. Preconcentration methods vary depending on the composition of marine sample extracts, the targeted glycolipid subclass, and the intended chromatographic method used in LC-MS.

The amphiphilic nature of glycolipids permits the application of different modes of LC separation, including normal-phase liquid chromatography (NPLC), reverse-phase liquid chromatography (RPLC), and hydrophilic interaction liquid chromatography (HILIC) [48,49]. NPLC and HILIC utilise interactions with polar head groups, while separation using RPLC is attributed to hydrophobic interactions with the lipidic tail group. In the recent years, HILIC has replaced NPLC for the analysis of polar lipid classes, due to its better compatibility with mobile phase solvents used in ESI-MS [48,50,51]. HILIC-MS/MS approaches are more common in polar lipid profiling studies [8,52,53]. Resolution of subclasses of GGLs (MGDG, DGDG and SQDG) [48,54], gangliosides [30,31], and even the configurational isomers of hexosylceramides (gluCer and galCer) [55] has been achieved using HILIC. Ionisable glycolipids such as SQDG and gangliosides can behave differently under different HILIC conditions, which requires the sample-specific optimisation of chromatographic parameters [48,56]. Alternatively, RPLC-based targeted analyses focus on the resolution of individual molecular species from each subclass like gluCer [57] and SQDG following their purification from crude sample matrices [46,47].

Understanding the lipid class composition of marine extracts/fractions is critical prior to the application of any LC-MS/MS method that is established for specific glycolipid determination. Thin-layer chromatography (TLC) can be used for the preliminary detection and quantification of target lipid classes in marine sample extracts. For subclass analysis, special approaches, like multistage sequential development TLC coupled with flame ionisation detection (FID), are required for GGLs [58,59,60]. TLC coupled with MS techniques are useful for the rapid characterisation of lipid structures without extensive sample processing [61,62], and can be harnessed for marine glycolipids analysis as a complementary approach to LC-MS/MS.

As well as targeted analyses of specific glycolipid subclasses, lipidomic investigations based on untargeted LC-MS/MS are generally aimed at the comprehensive characterisation of lipid molecular species belonging to various lipid classes/subclasses. Only a small number of glycolipid structures are identified by routine lipidomic studies compared to other polar lipids [9,15,36,63,64,65]. Improving resolution and reducing ion suppression is important for the enhanced detection coverage of glycolipids. Along with RPLC, other chromatographic approaches, such as supercritical fluid chromatography (SFC) and two-dimensional liquid chromatography (2D-LC) comprising HILIC-RPLC or SFC-RPLC, have been developed for the rapid resolution and comprehensive characterisation of lipid molecules [66,67,68,69,70]. Advancements in automated data processing software and LC-MS/MS structural databases have also been applied for the improved identification of glycolipids in lipidomic investigations [71,72].

This review summarises the analytical chemistry approaches applied for different subclasses of marine GGLs, cerebrosides and gangliosides, extracted from a range of marine organisms. Extraction and purification techniques relevant to glycolipid enrichment are discussed, along with the LC-MS/MS methodologies enabling the rapid resolution and improved identification of targeted glycolipid subclass and molecular species. Advances in TLC-coupled techniques are also discussed as both a supplementary (preliminary detection prior to LC-MS/MS) and complementary approach (TLC-MS) to the rapid characterisation of glycolipid subclasses.

## 2. Methods for the Extraction and Concentration of Marine Glycolipids

Marine glycolipids can be extracted and concentrated using a combination of biphasic, sequential, or solid-phase extraction (SPE), or column chromatography. Marine glycolipids can then be analysed either directly from the initial extracts or from the purified fractions that are enriched in polar lipids and/or glycolipids.

### 2.1. Methods of Glycolipid Extraction

Biphasic solvent extraction results in most glycolipids being partitioned into the organic phase [52,73,74,75], while leaving polar gangliosides in the aqueous phase [76,77,78,79], requiring recovery from both phases for comprehensive characterisation. Total lipid extraction methods, such as Bligh and Dyer [80] and Folch [81], have been widely applied to extract glycolipids [22,47,57,74] for LC-MS analysis, but often result in the enrichment of pigments that interfere with the analysis [47,82,83].

The multistep and laborious nature of conventional chloroform:methanol-based extractions has led to the development of alternative rapid methods, such as methyl-tert-butyl ether (MTBE):methanol [84], butanol:methanol (BUME) [85], and hexane:isopropanol (HIP) [86]. The MTBE:methanol and BUME methods have been applied in several lipidomic studies [87,88,89,90], and are particularly amenable to automated extraction, as lipids are enriched in the upper organic phase, in contrast to the lower organic phase in the conventional chloroform:methanol extractions. Although MTBE:methanol extraction is reported to be as efficient as chloroform:methanol [84,87], its effect on polar lipid enrichment, including glycolipids, has not been studied in-depth. Similarly, the BUME method, which was originally developed for rapid extraction of lipids from animal tissues, has been shown to have recoveries of polar lipids similar to the Folch and MTBE methods [85]. Further investigation is required, considering its limited application to samples of marine-origin. The HIP method is reportedly selective toward non-polar lipids [91,92,93], which limits its application for glycolipid extraction. Different solvent systems used in the glycolipid analytical workflow is presented in Table 1.

Due to their polar nature, specific methods have been developed for highly polar glycolipids such as gangliosides. The method of Svennerholm and Fredman [76] has been widely adopted for the analysis of gangliosides. It is based on monophasic extraction using chloroform:methanol:water (4:8:3, *v*/*v*/*v*), followed by phase partitioning through the of addition of water and subsequent recovery from the aqueous phase using dialysis against water. The method has been applied to a variety of marine samples, with numerous ganglioside molecular species unique to marine sources being characterised [30,31,94], as discussed in Section 4.1. An alternative rapid extraction method using a monophasic solvent mixture composed of chloroform:methanol:water (1:2:0.74, *v*/*v*/*v*) has been reported by Lydic et al. [95]. The extraction technique does not involve specific purification/phase partitioning for targeted analyses, making it suitable for lipidomic studies.

In addition to conventional solvent extraction approaches, the use of advanced methods, such as pressurised liquid extraction (PLE), ultrasonic-assisted extraction (UAE), and supercritical fluid extraction (SFE), have been more recently applied to the quantitative recovery of both neutral and polar lipid classes [96,97,98]. These methods can enhance extraction efficiency while using greener solvents, such as ethanol or supercritical CO_2_ (scCO_2_), and have been applied to a range of sample matrices of marine origin, including microalgae, seaweeds, and seafood species [99,100,101].

Senorans et al. extracted microalgal lipids using PLE and UAE techniques without the use of enzymatic pretreatment, and recovered higher levels of glycolipid (24.8% and 21.7% of total lipids, respectively) than the conventional Folch or Bligh and Dyer methods (13.9% and 8.3% of total lipids, respectively) [96]. Melo et al. showed that UAE can enhance the yield of polar lipid in ethanolic extracts, which otherwise tend to be lower than chloroform:methanol-based methods [16]. In the study, the yield of GGLs reported by LC-MS/MS of ethanolic extracts treated by UAE were comparable to chloroform:methanol-based extractions. A recent investigation of UAE technique on microalgal lipid extraction was reported by Puhringer et al. using a glycolipidomics approach [98]. The yield of total lipids by UAE using ethanol, without enzymatic pretreatment, was reported to be twice the yield of Folch extraction. Glycolipid subclasses, MGDG and DGDG, shared a significant fraction of lipids yield, constituting 17% and 12% of total lipids, respectively [98]. Ultrasonics can increase extraction yield through more effective membrane disruption [102], as can the increased temperature or pressure conditions of PLE [100].

SFE based on scCO_2_ has been investigated for marine lipid extraction, primarily targeting neutral lipids. However, the addition of polar co-solvents has also aided in the extraction of more polar compounds [101,103,104,105,106]. Servaes et al. more than doubled the total lipid yield from microalgal biomass by adding 30% ethanol as a co-solvent in scCO_2_ extraction [93]. The increased yield was primarily attributed to the enhanced extraction of glycolipids and phospholipids. Although the efficacy of scCO_2_ can be enhanced by the addition of a co-solvent, overall extraction efficiency was shown to be significantly lower than PLE with ethanol, which yielded lipids as high as 49% of dry biomass, with more than 10% belonging to glycolipid classes [93].

Pretreatment steps are important for effective scCO_2_ extraction. Washing the biomass with water and then freeze-drying can result in hypo-osmotic shock and cell disruption, enabling the more efficient mass transfer and increased yield of scCO_2_ extracts. Elst et al. found that scCO_2_ extraction of such a pretreated sample with 15% ethanol co-solvent resulted in a threefold increase in total lipid yield, reaching 23.1% of dry biomass, an amount comparable to that obtained with chloroform:methanol [97]. Additionally, pretreatment impacted lipid class extraction with, for example, increased DGDG versus MGDG in pretreated samples. The use of a porous solid support, such as diatomite, in scCO_2_ extraction was reported by Yang et al., resulting in an 81% enhancement in the efficiency of total lipid extraction and a 98% increase in the extraction of γ-linoleic acid-containing glycolipids [107]. This was associated with an increased bulk volume and surface area, resulting in the better dispersion of algal biomass and more effective solvent penetration. Under optimal conditions (solid materials-to-algal biomass ratio of 1:2 (*w*/*w*); solid materials’ water content of 60% (*w*/*w*); ethanol-to-biomass ratio of 3:1 (*v*/*w*); temperature of 40 °C; pressure of 41 MPa; CO_2_-to-mass ratio of 2.0 mL/g/min), the yield of γ-linoleic acid reported (34.52%) was significantly higher than that obtained using the Bligh and Dyer method, which yielded only 23.95% of total fatty acids [107]. Given that γ-linoleic acid predominantly occurs in galactolipids (MGDG, DGDG, and SQDG) and that LC-MS/MS analysis reported the presence of 27 molecular species of galactolipids in the same extract, the study showed that GGLs can be extracted using scCO_2_-ethanol extraction.

ScCO_2_ extraction methods targeting marine cerebrosides and gangliosides have not yet been widely investigated. Ji et al. reported the extraction of gangliosides from pig brain with scCO_2_ and ethanol as a co-solvent [108]. The method resulted in yields of only 15% of total gangliosides when acetone-precipitated brain extracts were subjected to scCO_2_ extraction with 75% ethanol at a 1:3 (*w*/*v*) solid:solvent ratio for 4 h at 70 °C and 30 Mpa. Similarly, Xu et al. applied scCO_2_ extraction to sea cucumber cerebrosides, resulting in only partial extraction [109]. In general, scCO_2_ extraction results in lower yields than those obtained using conventional methods. A summary of advanced lipid extraction techniques showing the enrichment levels of glycolipids compared to traditional methods is shown in Table 2.

Dimethyl ether (DME) is a useful solvent for the extraction of glycolipids due to its low boiling point (−24.8 °C), medium polarity, and relatively low toxicity. DME has been applied to the extraction of lipid compounds of varying polarities from marine tissues containing high amounts of water [106,110,111,112,113,114,115,116]. The effectiveness of DME extraction of microalgal and seafood samples have been previously reviewed [106,115]. Recently, Morel et al. reported that sub-critical DME is an effective alternative to scCO_2_ for the enhanced extraction of phospholipids from marine material, including Greenshell^TM^ mussel (*Perna canaliculus*), hoki (*Macruronus novaezelandiae*), and jack mackerel (*Trachurus* spp.) [42]. The advantage of DME over scCO_2_-based extraction is primarily related to its higher polarity. DME has the ability to form H-bonds with water present in wet samples so that the water acts as a co-solvent for extracting polar lipids, in contrast to the hydrophobic nature of scCO_2_ [117]. Studies on the use of DME for glycolipid extraction have reported low efficacy for glycolipids like GGLs and cerebrosides. In non-marine material (pteridophyte; fern), Nekrasov et al. found that DME appeared to extract most of the neutral lipids [110]. They also reported a higher abundance of GGLs and cerebrosides in the residual material left after sequential extractions using DME followed by DME and ethanol. Another study by Wang et al. indicated that total lipid yields could be enhanced by the addition of ethanol and acetone in DMEs in microalgal samples [118]. Similarly, Catchpole et al. highlighted the potential of sequential extraction with scCO_2_, DME, and DME, with ethanol as a co-solvent, for the enrichment of polar lipids [117]. There are only a few studies using DME to target specific marine glycolipids, as more polar glycolipids remain in the residual material without the use of more polar co-solvents. The integration of sequential extraction steps using DME and polar co-solvents is a promising technique for glycolipid isolation and concentration.

Chloroform:methanol-based biphasic extractions remain the most widely applied and efficient extraction methods for marine glycolipids. Examining the efficacy of alternative methods for glycolipid enrichment remains an important area of investigation, as advanced lipid extraction techniques using greener and less toxic solvents continue to evolve.

### 2.2. Methods of Glycolipid Purification

Marine lipid extracts are often complex in nature, containing pigments, phospholipids, and other metabolites that co-extract with glycolipids. Subsequent analyses of extracts using TLC and LC-MS can lead to the co-elution of these molecules with less abundant glycolipid species, impeding identification [43,44,45]. To overcome this problem, methods of purification need to be tailored to each sample type prior to analysis. Due to differences in glycolipid charge states and polarity, purification methods specific to each glycolipid type are essential.

GGLs can be purified using normal-phase flash chromatography with solvents of varying polarity, such as chloroform, acetone, and methanol, where the acetone fraction is analysed for glycolipids [74,75,93]. Depending on the sample composition, phospholipids can co-elute with more polar glycolipids, such as DGDG and SQDG. Therefore, these polar lipids can spread along the acetone and methanol fractions [73,119], limiting accuracy in subsequent analysis. Most analyses of macroalgal glycolipids have reported the use of acetone and methanol (e.g., 9:1, *v*/*v*) to collect total GGLs (MGDG, DGDG, and SQDG) in one fraction following the removal of neutral lipids and pigments [52,120,121,122]. In some cases, slightly alkaline solvents are used to elute acidic glycolipids like SQDG [123,124]. In cases where co-eluting pigments (chlorophyll) interfere with glycolipid analyses, techniques, such as selective de-greening using centrifugal partition chromatography, could be examined, as discussed by Kim et al. [125].

GSLs are usually purified from crude lipids following a mild alkali treatment to remove glycerolipids and phospholipids [126]. Obtaining a pure total GSL fraction using this method can be challenging, as some alkali-resistant phospholipids can remain, while some alkali-labile GSLs with O-acyl and O-acetyl groups can be lost [79,126,127]. Chromatographic methods developed in earlier studies were based on the separation of derivatised glycolipid products, modified by acetylation prior to purification and de-acetylation following purification [79,128]. These methods are laborious and limit quantitative recovery. Moreover, the separation of intact GSLs using traditional silicic acid chromatography can be challenging due to the similar chromatographic behaviour of phospholipids. Recent developments in column chromatographic purification of GSLs use stationary phases like titanium dioxide (TiO_2_) [129] or zirconium dioxide (ZrO_2_) [130] sorbents. These selectively bind phospholipids, enabling efficient separation of intact GSLs without derivatisation. These stationary phases are primarily used for the separation of neutral GSLs in extracts that also contain alkali-resistant sphingomyelin—glycerophospholipids possessing ether bonds, alkyl, or alkenyl chains. GSLs with glycan chains longer than tetraglycosylceramides, as well as those containing additional hydroxy fatty acids or gangliosides, can be strongly retained on these phases limiting their quantitative recovery [130].

Unlike neutral GSLs, acidic GSLs/gangliosides can be recovered from the aqueous phase of biphasic extraction systems and then purified by SPE using reverse-phase C8 or C18 silica sorbents to remove salt and sugar contaminants [30,31,56,94,131,132]. Negatively charged sialic-acid-containing gangliosides have affinity toward anion exchange functionalities like diethylaminoethyl (DEAE), enabling the enrichment of target gangliosides [133,134,135]. Additionally, gel filtration chromatography can be applied to samples with low concentrations of gangliosides to remove low-molecular-weight contaminants [78,134]. As discussed in Leenders et al., a combination of both reverse-phase cartridges and gel filtration resulted in the quantitative recovery of gangliosides up to 67% from blood plasma samples [78]. A method for ganglioside extraction from marine samples reported by Svennerholm and Fredman has been applied by multiple investigators, with most studies using reverse-phase cartridges for subsequent purification [30,31,94]. In the original Svennerholm and Fredman method, brain gangliosides were purified quantitatively using dialysis against water, which improved ganglioside recovery relative to anion exchange/gel filtration media [76]. However, variations in sample source and composition of target gangliosides make these methods difficult to directly compare.

Khoury et al. reported a rapid purification method for gangliosides using LC-MS, which involved monophasic extraction and enrichment using specific lipid-class binding sorbents such as Phree^TM^ phospholipid removal cartridges (Phenomenex, Torrance, CA, USA) [45]. These cartridges improve the purification and detection sensitivity of gangliosides in LC-MS, compared to those extracts purified using C18 SPE cartridges. However, enhancements in sensitivity are dependent on the type of sample matrix. The method is useful for samples with high phospholipid levels, which can cause the ion suppression of gangliosides in LC-MS if not removed.

These different purification techniques are usually supplemented with qualitative TLC staining methods targeted to specific glycolipid subclasses. This enables the elution solvents used in the chromatography to be systematically modified based on extract composition. Method efficacy is tested by quantifying the glycolipids in enriched fractions using measurements of either lipid-bound sialic acid, using resorcinol for gangliosides [27,76,136], or orcinol/anthrone-sulfuric-acid-based colorimetric assays for neutral GSLs and GGLs [137]. These sugar-based assays only provide an estimation of glycolipids, since quantification is based on an adjusted galactose standard curve prepared by multiplying equivalent galactose content by a factor of 2.8 [138,139,140,141]. Accurate quantification requires the use of TLC or LC coupled with specific detectors, where each glycolipid subclass/molecular peak can be quantified based on the respective standards.

## 3. TLC-Based Detection Methods for Glycolipids

### 3.1. Qualitative TLC

TLC separation of glycolipid subclasses is visualised using carbohydrate-specific staining reagents. Orcinol–sulfuric acid is commonly used for detecting the neutral sugars found in GGLs and GSLs, whereas resorcinol–hydrochloric acid specifically detects the sialic acid-containing sugars present in anionic glycolipids like gangliosides [136,137]. Non-specific and non-destructive visualisation stains, such as primuline reagent [128] or iodine vapour [142,143], can also be used to identify glycolipids at equivalent retardation factor (R_f_) values by comparison with glycolipid standards. Retention in normal-phase TLC is mostly determined by the interaction of the polar sugar head groups with the stationary phase, resulting in the resolution of individual subclasses. Some subclasses have slightly different R_f_ values to that of commercial standards, depending on the sample characteristics, chain length, and unsaturation present in the lipidic tail. Additional methods of identification, such as TLC coupled with MS or immunostaining, may be required [144,145]. Immunostaining methods using antigen–antibody-binding assays can be applied to detect specific oligosaccharide groups and are useful for the identification of ganglioside subclasses [145,146,147].

Marine-derived GGL-rich fractions can be resolved using TLC into MGDG, DGDG, and SQDG using a solvent mixture of chloroform, methanol, and water, for example, in a ratio of 65:25:4, *v*/*v*/*v*, respectively [60,73,75,148]. Likewise, neutral GSLs such as glycosylceramides can be separated into individual subclasses using the same solvent mixture at varied proportions/polarity, for example, chloroform, methanol, and water in a ratio of 60:35:8, *v*/*v*/*v*, respectively [128,136,145,149]. Separation of acidic GSLs like gangliosides can be improved by the addition of an aqueous salt solution (e.g., 0.2% (*w*/*v*) CaCl_2_) in a chloroform, methanol, and water-based developing solvent [27,128,142,145,150].

Approaches like multiple development TLC and 2D-TLC have been applied as rapid methods to resolve specific subclasses of GGLs (MGDG, DGDG, and SQDG), cerebrosides, and gangliosides from complex lipid extracts [58,60,151,152,153,154,155]. The method involves multiple sequential developments of a TLC plate in the same direction using solvent mixtures of increasing polarity. In 2D-TLC, a second development is carried out in a direction orthogonal to the first to enable the separation of the different lipid subclasses present at low levels. The 2D-TLC method has been applied for resolving gangliosides extracted from animal tissues, including fish samples [134,151,156,157]. Unlike mammalian tissues, relatively polar and distinct gangliosides structures, such as O-acetylated and lactonized gangliosides, were detected in fish extracts [156,158,159].

Table 3 summarises key TLC approaches applied to samples (glycolipid fractions or crude lipids) to resolve major subclasses of glycolipids.

TLC plates with precoated and chemically modified stationary phases can selectively alter the retention of glycolipids with varying polarities and charged states [142,160]. TLC plates modified with aminopropyl siloxane, which offers weak anion exchange properties, can alter selectivity towards acidic glycolipids like SQDG, sulphated hexosylceramides, and gangliosides. Similarly, TLC plates impregnated with boric acid or other inorganic ions can alter the chromatography of compounds that possess H-bonding functional groups [160]. Improvements in the chromatographic resolution of phospholipids [161,162,163] and carbohydrates [164] have been investigated, but reports on specific glycolipid classes are limited to studies reporting on improvements in the separation of GSLs and lyso-GSLs using boric-acid-impregnated TLC plates [165].

### 3.2. Quantitative TLC-Coupled Detection Methods

#### 3.2.1. TLC-Densitometry

TLC-densitometry is generally applied for comparative estimations of lipid classes/sub-classes present in different samples following derivatisation with specific reagents [155,166,167,168]. The methods and application of TLC-Densitometry in the analyses of lipids have been reviewed previously [142,169]. Densitometric scanning utilises ultraviolet (UV) absorption or fluorescence emission from the separated compounds on TLC plates [169,170]. Most glycolipid species are non-UV active, and therefore require derivatisation to incorporate a chromophore or fluorophore prior to densitometric scanning [35,128,171].

Primuline is the most common stain used in fluorescence densitometry, as it is highly sensitive and does not form covalent bonds with compounds, allowing for non-destructive visualisation. It is one of the most sensitive TLC techniques for general lipid detection, including glycolipids and phospholipids [128,142,169,172,173,174]. UV-scanning densitometry requires the derivatisation of glycolipids with sugar or sialic acid-based staining reagents, such as orcinol–sulphuric acid [169,174] or resorcinol–hydrochloric acid [175,176]. Another specific stain, Azure A, is a cationic methylated thiazine prepared in sulfuric acid, and is used for the separation and quantification of sulfatides or sulphated glycolipids [177]. TLC-densitometry using immunostaining methods are developed for GSL/ganglioside detection, where specific ligands (anti-GSL antibodies) are used to detect the lipid-bound oligosaccharides of specific structures [144,150,178]. The application of TLC-densitometric techniques for the evaluation of glycosphingolipids of higher animals is relatively well established, but the use of these methods remains limited in marine samples, partly due to a lack of marine-derived glycolipid standards [155].

#### 3.2.2. TLC-FID

TLC coupled with FID (Iatroscan^TM^) is routinely used for qualitative and quantitative analyses of different lipid classes [179,180,181]. It has broad applicability to a wide range of compounds, including molecules with low volatility, and has been applied to marine lipid analysis since the 1980s [182,183]. The method enables the analysis of intact compounds without the requirement for chemical derivatisation, and while the method destroys the sample during detection, only a small amount of sample is required for analyses [60,179].

Depending on the complexity of the target sample matrix, multiple-development TLC-FID techniques has been applied for optimising the resolution and accuracy of quantification for several lipid classes, including GGLs. Without prior fractionation, single-development TLC-FID analyses of marine lipids result in glycolipids co-eluting with pigments, monoacylglycerol (MAG), and phospholipids as acetone-mobile polar lipids [60,184]. Multiple-development TLC coupled with partial-scan FID has made it possible to resolve the major GGL subclasses (MGDG, DGDG, and SQDG) without fractionating total lipid extracts [60]. This method was developed after successful earlier experiments by Parrish and Ackman, which resolved several neutral lipid subclasses [183], as well as 11 other lipid classes using a similar approach [182]. It was shown that TLC development in the solvent mixture based on chloroform (e.g., chloroform:acetone (3:2, *v*/*v*)) resolved pigment classes from MGDG, whereas the solvents based on acetone (e.g., acetone:formic acid (49:1, *v*/*v*)) enabled resolution of more polar GGL subclasses, such as DGDG and SQDG from phospholipids [60]. These results were supported by Striby et al., who reported a similar resolution of MAGs, pigments, and GGLs (MGDG and DGDG) from phospholipids after two successive TLC developments, which included seven minutes of development in 100% acetone, followed by 35 min of development in a solvent mixture composed of chloroform:acetone:formic acid (99:1:0.2, *v*/*v*/*v*) [59]. SQDG was not reported in the study. However, due to the similar anionic properties of SQDG and phospholipids, these two classes likely co-eluted. Gasparovic et al. reported a multiple-development approach optimised for visualising the separation of 18 lipid classes, including GGLs (MGDG, DGDG, and SQDG) from marine extracts, through sequential developments in seven different solvent mixtures with increasing polarities [58]. This method used solvents based on chloroform:acetone:formic acid (95:5:0.6, *v*/*v*/*v*) to improve the separation of pigments from glycolipids, and two successive TLC developments in acetone:chloroform (72:28, *v*/*v*) and acetone:chloroform:methanol:formic acid (33:33:33:0.6, *v*/*v*/*v*/*v*) enabled the resolution of the individual GGL subclasses.

These studies indicate that higher percentages of chloroform in the development solvent results in a better resolution between pigments and MGDG, while acetone-based solvents are required for the separation of both MGDG and DGDG. These methods are promising for resolving GGLs from complex mixtures, enabling rapid preliminary quantitation. Further optimisation may be possible if selective extraction/enrichment methods are developed for specific glycolipids of interest prior to analysis using TLC-FID. Reducing the pH of solvent systems can also improve selectivity toward more polar and anionic glycolipids, as the silanol layers are generally ionised in neutral-to-basic pH, which can repel the anionic analytes from the stationary phase. The applicability of TLC-FID for the rapid quantitation of glycolipids other than GGLs is absent in the literature, and yet it is potentially suitable for other types of glycolipids, such as cerebrosides and gangliosides, which are present in various marine tissue.

### 3.3. TLC-MS

TLC can also be coupled with MS for the direct molecular characterisation of lipid classes. MS facilitates the identification of structures based on the molecular mass and characteristic MS/MS spectra, in addition to the chromatographic R_f_ value obtained from TLC, so that individual compounds can be identified without purification [62,185]. TLC plates of smaller particle size (5–6 micron), also known as high-performance TLC, are used for TLC-MS. These plates are specifically suited for coupling with MS, as they have shorter development times, reduced solvent consumption, and lower sample diffusion. TLC-MS uses soft ionisation MS methods, such as matrix-assisted laser desorption ionisation (MALDI) and elution-based ESI-MS interfaces, to maximise the intensity of the parent ions [61,185,186]. MALDI results in the ionisation of target analytes from the TLC plate under vacuum, whereas elution-based ESI interfaces, such as desorption electrospray ionisation (DESI) [187,188,189] or liquid extraction surface analysis (LESA) [190], are carried out at ambient pressures. These techniques ensure the minimal fragmentation of analytes and rapid detection by reducing the need for extensive sample preparation. TLC-MALDI-MS and TLC-DESI-MS, specifically focused on polar lipid analysis, have been previously reviewed [187,191].

TLC-MALDI-MS uses predominantly UV-absorbing matrices, such as 2,5-dihydroxybenzoic acid (DHB), *p*-nitroaniline, and 9-amino-acridine (9-AA), for the ionisation of separated compounds on a TLC plate using a nitrogen laser source [191,192]. The primary advantage of TLC-MALDI-MS compared to ESI-MS is that it simplifies the interpretation of mass spectra by minimising the formation of complex adducts [191,193]. Despite the rapidity of analysis, TLC-MALDI-MS has a major disadvantage, as commonly used matrices like DHB can undergo photochemical reactions, which can interfere with the mass analysis of small molecules (mass < 2000 g/mol), while others may reduce sensitivity/resolution due to spreading of analytes on the TLC plates [192,194]. An alkaline 9-AA-based matrix is suitable for acidic GSLs, whereas acidic matrices are suitable for neutral GSLs, as they reduce background noise in the MS [192,195]. The application of MALDI-MS may be challenging for some glycolipids, such as sialic acid-containing gangliosides, which can suffer from in-source fragmentation due to the matrix type, laser source, and vacuum conditions [185,196]. Developments of TLC-MALDI-MS interfaces for complex lipids have been previously reviewed, particularly with regard to the use of advanced matrices, such as graphite-based materials, to enhance detection sensitivity with minimal fragmentation and lower background noise [192,197,198]. Cha and Yeung reported on colloidal graphite-based MALDI-MS, which significantly improved the detection of cerebrosides from total lipid extracts, resulting in the detection of an additional 14 cerebroside molecular species compared to general MALDI-MS [199]. Similarly, Hua et al. demonstrated that graphene nanoflakes reduced ion-suppression, providing enhanced sensitivity in the detection of lipid molecular species using MALDI-MS [200]. Significant improvements in detection sensitivity were also reported by Wang et al., where 65 lipid species were detected, including phospholipids and GSLs, when graphene oxide was used as a matrix compared to a DHB matrix (which enabled the detection of only 13 lipid species) [201]. These studies illustrate the potential use of graphene materials in the rapid analysis of lipid molecular species, while also enhancing detection sensitivity for extracts without the requirement for extensive sample workup.

Elution-based TLC-MS methods are similar to conventional methods where analytes are recovered from a developed TLC plate and analysed using ionisation at ambient pressure using ESI-MS, but the recovery process is automated [202]. Unlike MALDI, elution-based methods reduce artefact spectra. This is because no matrix or vacuum is required for ionisation, making it well-suited to the analysis of small molecules, including lipids [188,203]. In TLC-DESI-MS an organic solvent is electrosprayed onto the TLC plate, which causes the desorption or droplet pickup following ionisation under ambient conditions [204,205]. LESA-MS is another widely used ambient ionisation interface known to be well-suited for high-throughput analysis of lipids [202,206,207]. This technique enables rapid screening by combining an automated micro-liquid extraction system with nano-electrospray ionisation MS. Unlike DESI-MS, which requires higher temperatures to extract and ionise the analytes via droplet pickup from the TLC plate, LESA-MS independently samples and ionises analytes, thereby improving detection sensitivity while also preserving thermally labile compounds [206]. These studies are primarily focused on the improvement of analyte recovery through the optimisation of solvent selection for micro-extraction and point of sampling from the bands on TLC plates [185,208]. These ESI-based interfaces are reportedly better than MALDI for the analysis of polar analytes, including GSLs containing sialic acid residues [185].

The coupling of TLC with MS detectors can be useful to rapidly characterise a range of lipid compounds, including GGLs and GSLs, particularly those that are present in low abundance, since direct characterisation with minimal sample preparation reduces the possibility of sample loss and degradation. The development of the TLC-MS method enables rapid screening with enhanced spatial resolution and sensitivity for a range of lipid compounds [61,194]. Matrix selection is important for TLC-MALDI-MS analysis [186,209], whereas solvent selection and sampling position is important to enhance the detection sensitivity of complex lipids while using elution-based interfaces.

A summary of TLC-coupled techniques facilitating glycolipids characterisation is presented in Table 4.

## 4. LC-MS/MS Methods for the Analysis of Marine Glycolipids

In addition to TLC-coupled analytical techniques, improvements in LC-coupled MS/MS methodologies, with enhanced sensitivity and resolution, are useful for rapid structural characterisation of glycolipids. These methods require more sophisticated instrument setups and the use of columns containing stationary phases with advanced functionalities. LC-MS/MS methods for analysing glycolipids are specifically developed for each of GGLs, cerebrosides and gangliosides owing to their diverse structures and retention characteristics. In the LC domain, the detection of minor glycolipid species can be enhanced through fractionation/isolation of target subclasses prior to analysis, thereby reducing the possibility of isomeric and isobaric overlaps. Additionally, in the MS domain, characteristic MS/MS fragmentation profiles typical to specific glycolipid subclasses and the relative mobility based on shape, size, and conformation of a parent ion molecule, aids in strengthening the accuracy of structural identification. Four different forms of separation methods (HILIC, RPLC, SFC and 2D-LC) relevant to analyses of a range of glycolipid types are discussed in Section 4.1, Section 4.2, Section 4.3 and Section 4.4 and a brief insight in MS methods for advancing structural characterisation of glycolipids is discussed in Section 4.5.

### 4.1. HILIC-MS

HILIC has been used extensively as a separation mode in the LC-MS analysis of marine-derived polar lipids due to its proven advantages over NPLC [37,212]. This technique was first introduced in 1990 as a variant of NPLC [213], and it is suitable for the sensitive separation and analysis of polar compounds, such as sugars, amino acids, and complex polar lipids, when applied in LC-MS/MS [212,214,215]. The separation of these compounds can be challenging due to retention problems in reverse-phase sorbents and solubility issues in normal-phase eluents. To overcome these issues, HILIC uses stationary phases, similar to those used in NPLC, while operating under RPLC-like aqueous mobile-phase conditions [216,217,218]. The mechanism of HILIC is based on mixed-mode retention, which includes hydrophilic partitioning, hydrogen bonding, and electrostatic/dipole–dipole interactions. The careful optimisation of mobile-phase pH and ion-pairing reagents is required to control the retention and selectivity of charged polar analytes under various HILIC conditions [212,217,219].

The subclass-level resolution of lipids resulting from HILIC enables the relative quantification and semi-quantification of individual lipid species belonging to each subclass, as those with the same headgroup tend to have similar ionisation efficiencies [220,221]. The accuracy of relative quantification is usually improved through normalisation of the analyte peak intensity with structurally similar internal standards of similar polarity and retention characteristics [222]. This quantification approach has been applied to samples where the availability of standards is limited, such as glycolipids of marine origin [14,223,224].

Analytical methods based on HILIC-MS are well established for the analysis of acidic GSLs (e.g., gangliosides) [27,30,31,56,225]. Under HILIC conditions, the separation of gangliosides is based on the number and nature of the sugar head group and sialic acid residues, and has been effective for the baseline resolution of the major subclasses of gangliosides, GM, GD, GT, and GQ, present in animal samples [56,225]. A HILIC-MS method for the analysis of mammalian gangliosides was first reported by Fong et al., where eight different subclasses of gangliosides, GM1, GM2, GM3, GD3, GD1a, GD1b, GT1b, and GQ1b, were resolved from rat brain extract [225]. In the study, a gradient method based on an acetonitrile and ammonium acetate buffer was used on an aminopropyl HILIC column at pH 5.6, which enabled the quantification of those subclasses, along with the determination of the relative abundance of individual molecular species. These amino HILIC columns are specifically designed to offer added ion exchange capability, and appear to be well-suited for acidic compounds [212]. The method reported by Fong et al. may have wider applicability for other ganglioside subclasses, such as those containing N-glycolyl neuraminic acid (Neu5Gc) and O-acetylated gangliosides. Given the distinct nature of glycan head groups across different animal species, the careful optimisation of existing HILIC methods is required for the resolution of ganglioside subclasses. Up to 200 subclasses of gangliosides have been reported from animals [27,28] with varying sugar unit and sialic acid residue compositions. Within each of these subclasses, differences in the chain length, unsaturation, and hydroxylation of the ceramide unit exist, which can also impact HILIC separation. This was shown in a study by Hajek et al., where a change in the C-chain length of the ceramide tail reduced the retention of individual molecular species of the GD1a subclass [56]. The primary difference between gangliosides of marine origin and those from other organisms is the type of sialic acid sugars present. Non-marine residues are composed primarily of N-acetyl (Neu5Ac) and N-glycolyl (Neu5Gc) neuraminic acid as sialylated sugars [31], while those from marine sources contain additional sulphated sugars or fucose that are unique to each species [30]. More than 50 different sialic acids have been described previously, including unique structures like KDN (2-keto-3-deoxy-D-glycero-D-galacto-nonulosonic acid) and C11-hydroxylated Neu5Gc, which are present in the gangliosides found in lower vertebrates and some invertebrates like sea urchin and sea cucumber [31,226,227].

The method developed by Fong et al., 2009 has been applied for the analysis of gangliosides extracted from fish, poultry, and beef in a 2016 study [94]. Ganglioside molecular species belonging to the subclasses GM3, GD3, GD1a, GD1b, and GT1b were identified and quantified as a total subclass peak, as described in Table 5. The study reported a higher relative abundance of GM_3_ subclass, whereas a lesser abundance of GD and GT subclasses was reported in all samples tested [94]. The result appears to be different from the earlier TLC-based studies on fish gangliosides [156,158], which indicated the presence of highly polar gangliosides with multiple sialylation and additional modification such as O-acetylation, as discussed in Section 3.1. These differences in ganglioside profiles can be attributed to the type of fish tissues being used in these studies, with the former analysing the fillets as a source of dietary gangliosides and the later extracting gangliosides from brain tissues. More studies are required to characterise ganglioside molecular diversity and abundance across different fish tissues.

Another HILIC-MS method for the analysis of complex vertebrate gangliosides was described by Hajek et al., where 145 ganglioside species were characterised from 19 subclasses, where GM1a, fucosylated GM1a, GT1b, and the isomers GD1a and GD1b were resolved at baseline [56]. In the study, the use of 10 mM ammonium acetate at mobile-phase pH of 6.1 was applied to the resolution of isomers GD1a and GD1b. Additionally, the formation of one ionic form of ganglioside subclasses was important in HILIC-MS, as the subclasses with multiple sialic acids, such as GD and GT, have multiple charged forms, affecting the chromatographic peak shape and resolution. A pH range of 5–10 was optimal for reducing the chromatographic peak tailing associated with different ionic forms of each subclass [56]. Although this method was applied to ganglioside analysis of terrestrial animals, it is applicable to samples of marine origin, given the occurrence of highly polar gangliosides in marine species.

In addition to vertebrate fish gangliosides, the HILIC method developed by Fong et al., 2009 has been systematically optimised and applied to the characterisation of sea urchin and sea cucumber gangliosides [30,31], as highlighted in Table 5. Ma et al. applied HILIC-MS to identify sea urchin gangliosides, which were distributed among 14 subclasses of mono-, di-, and tri-sialo groups (GM4, GD4, and GT4) [31]. These gangliosides consisted of unique sialic acid features such as Neu5Ac, Neu5Gc, or KDN, alongside modifications using substituents like sulphonic acid. Similarly, Wang et al. applied the HILIC-MS method to characterise 17 subclasses of sea cucumber gangliosides that had multiple sialylations in the sugar head (up to penta-sialo groups) together with characteristic sulphate, fucose, and inositol phosphate substituents [30].

As with the ganglioside analysis, HILIC-based separation methods have also been applied to LC-MS/MS structural characterisations of GGL subclasses (MGDG, DGDG, and SQDG). However, these GGLs are frequently detected as a part of total polar lipids (glycolipids, phospholipids, and betaine lipids) with limited specificity [8,15,52,53,64,65,121,140,232,233]. The detection of less abundant GGL subclasses occurring in marine samples, such as amino modified, lyso-, and acylated forms of GGLs [5,237], can be complicated by their co-elution with other molecular classes. HILIC-based analysis of GGLs as a separate lipid class distinct from other polar lipids has not been previously reported, which can be attributed to the inherent difficulty in isolating total GGLs in a single fraction where the co-elution of phospholipids and pigments occurs. This is where specific purification method/s developed for phospholipid and pigment removal could assist in a more targeted HILIC-based analysis of GGL subclasses.

There are only a few HILIC-based methods reported that specifically target neutral GSLs/cerebrosides subclasses. The two major subclasses of cerebrosides, gluCer and galCer, found in living organisms [21,238], are often detected/co-eluted as a single hexosyl ceramide peak, along with other polar lipids in HILIC-based lipidomic studies [54,70,239]. A few studies have reported the separation, detection, and quantification of these two subclasses using the HILIC-MS method for biological samples [55,240,241]. As reported by Nakajima et al., a HILIC zwitter ionic column was sufficient for the resolution of two distinct configurational isomers of hexosylceramides (gluCer and galCer), along with the additional separation based on the chain length and level of hydroxylation of the ceramide tail (fatty acid and spingoid base) [55]. Although this analysis is based on the sphingolipids extracted from mammalian brain, its capability to detect minor species of gluCer (which is 1/350 times less abundant than galCer) illustrates its potential for analysing a range of minor cerebroside subclasses from marine samples.

The nature of the stationary phase functionality determines the ability of HILIC columns to retain and separate glycolipids. A conventional bare silica column retains analytes primarily through hydrophilic partitioning and ion exchange interaction and hydrogen bonding [214,218]. This column has been used predominantly for profiling polar lipids from marine algae, including GGLs (MGDG, DGDG, and SQDG) and GSLs (hexosylceramides) and phospholipids (Table 5) [8,52,53,64,65,120,121,122,232,233]. Despite the ability to detect and characterise several glycolipid molecular species, the chromatographic resolution of individual GGL subclass peaks from the rest of the polar lipids remains suboptimal [52,53,232]. The satisfactory baseline resolution of individual GGL subclasses, like MGDGs, DGDGs, and SQDGs, has been achieved only in a small number of studies [48,54]. The HILIC-MS method used by Okazaki et al. for lipidomic assessment of the plant *Arabidopsis* resulted in the detection of distinct peaks of GGL subclasses (MGDG, DGDG, and SQDG), along with a range of other phospholipid subclasses [54]. In the study, a gradient elution method was applied on a diol-based silica column with a solvent mixture composed of acetonitrile, methanol, and water buffered with 0.2% ammonium formate at pH 5.9 for optimum resolution and detection of these lipid classes. Anesi and Guella also highlighted the efficacy of a diol-based silica column for the distinct resolution of GGL subclasses (MGDG, DGDG, and SQDG) from other polar lipids [48]. The separation and analysis of polar lipid classes has been validated using green alga (*Jaoa bullata*), marine dinoflagellate (*Peridinum cinctum*), and a terrestrial plant (*Vitis vinifera* cv. *corvina*). The retention of ionisable/negatively charged polar lipids appeared to be highly dependent on the pH and concentration of buffer additives such as ammonium acetate and formate [48,242]. The enhanced chromatographic resolution can be attributed to both the choice of mobile phase and the use of a diol-phase column, which is designed to minimise unnecessary ionic interactions while allowing for hydrogen bonding and hydrophilic partitioning effects to be the primary modes of HILIC retention [212,218,243].

Cífková et al. compared different HILIC column chemistries for the degree of resolution and peak shape in the analysis of acidic lipids [239]. A silica hydride column resulted in the superior chromatography of polar lipid classes, including neutral GSLs (hexosylceramides) and acidic phospholipids. These columns have silanol (Si-OH) groups replaced by hydride (Si-H) bonds to eliminate/reduce ionic interaction sites that lead to peak tailing and/or the possibility of irreversible adsorption [214,218]. Cífková et al. reported that the improved chromatography of acidic lipids is associated with both the stationary phase characteristics and control of pH and buffer concentration.

Several HILIC-MS methods targeting the analyses of polar lipids, including glycolipids, have been developed. Although most of these methods are based on non-marine samples, they appear to be applicable to a range of biological samples, including those of marine origin, due to the structural similarity of the head groups of most glycolipids. The systematic optimisation of these methods is likely to be required to resolve glycolipids from other polar lipids and enhance the detection capabilities of minor glycolipids with distinct sugar heads.

### 4.2. RPLC-MS/MS

Reverse-phase chromatography is widely used in LC-MS/MS (RPLC-MS/MS) and has been applied to the rapid lipidomic profiling of marine extracts from samples such as algae [14,90], invertebrates [229], and marine fish [231]. Although RPLC-MS/MS studies have detected some glycolipid structures, the number and types of glycolipids reported remain low compared to other polar lipids.

Cutignano et al. reported an RPLC-MS/MS method for profiling marine microalgal lipids using a biphenyl column [90]. The method enables the detection of several GGL molecular species (5–7 MGDGs, 3–7 DGDGs, and 2–5 SQDGs) from five species of marine microalgae (Table 5). In contrast to the routinely used C8 and C18 columns, the biphenyl stationary phase improved the resolution between galactolipids/SQDG and phospholipids subclasses, as well as among molecules differing in degree of unsaturation. The method did not require pH adjustment or the use of buffer salts, which prevented the formation of complex adducts in ESI-MS, thereby reducing ambiguity in the identification of lipid species.

Another RPLC-MS/MS method for analysing the total lipids extracted from marine fish samples was reported by Wang et al., where more than 700 lipid species were identified from 12 molecular subclasses [230]. Despite the ability to resolve a wide range of lipid species, only six molecular species of hexosylceramide glycolipids were detected from one of the marine fish samples (*S. maximus*) used in the study. To resolve total lipid molecular species, a gradient-elution RPLC C18 method was applied using mobile phases composed of isopropanol, acetonitrile, and water, with 0.1% formic acid as the eluent additive and 5 mM ammonium formate as the buffer [230]. The same method was adapted with minor modifications by Wang et al. in a 2022 study to analyse lipids from three seaweed species, where several molecules of MGDG (30–66 molecules), DGDG (20–45 molecules), acylated MGDG (19–121 molecules), acylated DGDG (6–35 molecules), and hexosylceramides (1–9 molecules) were identified [14]. Likewise, Li et al. reported an RPLC method coupled with triple time of flight (TOF)-MS/MS for the comparative analysis of lipid profiles among four edible shellfish species [228]. The study detected 4–16 molecular species of hexosylceramides with a much lower abundance (18.5–95.5 nmol/g) compared to other subclasses of phospholipids and sphingolipids. Hu et al. applied the same method for analysing complex marine lipids extracted from eight echinoderm species [229]. A total of 961 lipid molecular species were reported in the study, with the majority (76.83%) belonging to the phospholipid class (Table 5). SQDG (4–8 molecules) and hexosylceramide (39–73 molecules) were the only glycolipids present in detectable amounts [229].

A recent lipidomic study on marine fish oils was reported by Windarsih et al., where nearly 1000 lipid molecular species were detected using RPLC coupled with high-resolution Orbitrap-MS [231]. The study reported limited detection of glycolipids, including hexosylceramides, MGDG, and SQDG, with stark differences in detection sensitivity between the two ESI modes, with the positive ESI mode favouring the detection of neutral lipids classes. Additionally, the limited detection of glycolipids in this study can also be attributed to the use of the non-polar solvent *n*-hexane, used for the extraction of lipids [231]. These lipidomic-type studies have been useful for determining the types and abundance of some structurally diverse marine lipids found in a range of sample sources, although more comprehensive investigations are still required for identifying glycolipids from less abundant sample sources. In additional to lipidomic investigations, RPLC-based separations are also used for targeted analyses of certain glycolipids, such as cerebrosides [26,57,235,244], GGLs [47], and gangliosides [135], extracted from a range of marine-derived sample sources (Table 5).

Analysis of marine cerebrosides using RPLC coupled with Ion Trap-MS/MS was reported by Xu et al. [235], where 12–52 glucosylceramide structures were identified from three sea cucumber species (Table 5). The study applied a simple isocratic elution on a C18 column to resolve the cerebroside molecules, although the separation of cerebrosides from other lipid classes was required prior to analysis using LC-MS/MS. Jia et al. developed another RPLC-MS/MS method coupled with quadrupole time of flight (QTOF)-MS for the analysis of sea cucumber cerebrosides, resulting in the identification of 89 molecular species of cerebrosides from sea cucumber extracts [57]. Despite the similar chromatographic conditions under the isocratic elution mode, the resolving power and detection capability was significantly enhanced compared to the earlier study by Xu et al. because of the more sensitive QTOF-MS instrumentation. Additionally, the sample pretreatment and cerebroside purification methods used by Jia et al. probably enhanced the sensitivity of detection. Yamaguchi et al. applied isocratic elution RPLC coupled with an evaporative light-scattering detector and ion trap-TOF-MS/MS for analysis of starfish cerebrosides [26]. More than 23 cerebroside molecular peaks, including several structural isomers, were reported, with major differences in molecular diversity and abundance across various body parts of starfish (Table 5). In all of these studies, the RPLC method was coupled with MS to rapidly resolve and identify important structural variations in the hydrophobic ceramide tails (LCB and fatty acid groups). Marine cerebrosides consisting of triene groups in LCBs have potential cosmetics and nutritional applications due to their epidermal barrier [245,246] and skin hydration properties [5,238,247,248,249]. However, analyses of marine cerebrosides remain limited to a few invertebrate samples, partly due to the minimal application of modern RPLC-MS/MS methods for rapid separation and structural characterisation.

RPLC analysis of GGLs (MGDG, DGDG, and SQDG) can be carried out separately [47,250] or together [123,251,252] using C8 or C18 chromatography. Zábranská et al. optimised RPLC coupled with ESI-MS/MS separately for each galactolipid subclass isolated by TLC, where individual molecular species of MGDG and DGDG were resolved based on their C-chain length and level of unsaturation [250]. Similarly, Fisher et al. reported an RPLC-ESI-MS/MS method for identifying and quantifying sulfoglycolipids from marine and non-marine samples, where this GGL (SQDG) was purified using SPE prior to analysis [47]. Using an MS/MS fragmentation approach, the study optimised precursor–product transitions for multiple reaction monitoring (MRM) of individual sulfolipid molecules, enabling their sensitive and reliable quantitation. A study by Körber et al. followed a similar approach to analyse GGLs from a terrestrial plant sample, using RPLC-MS/MS to assess the relative abundance and molecular diversity of two separate fractions that contained galactolipids (MGDG and DGDG) and sulfolipids (SQDG), respectively [123]. These studies indicate that baseline resolution of individual molecular peaks can be achieved if the targeted GGL subclasses are purified prior to analysis.

Analysis of total gangliosides using reverse-phase chromatography has remained challenging due to inter-subclass co-elution of molecular species [35,253,254]. However, in targeted analysis, reverse-phase retention time, MS/MS fragmentation, and MRM can assist in the accurate identification and quantification of ganglioside molecular peaks [135,255,256]. As discussed by Cong et al., RPLC remains a preferred approach for the detection of trace ganglioside molecules of varied ceramide moieties within the same subclass [236]. The study reported an RPLC method specific to monosialogangliosides from sea urchins that consisted of sulphated and non-sulphated glycan heads and both Neu5Gc and Neu5Ac types of sialic acids (Table 5). A targeted MS/MS and MRM approach was applied in both positive and negative ESI mode for the identification of LCBs and sialic acid groups present in individual molecules of GM gangliosides [236].

### 4.3. SFC-MS

SFC-MS has been applied widely for lipid analysis [66,67,257,258,259,260,261]. SFC-MS uses scCO_2_ as a mobile phase, along with organic modifiers such as methanol for separating compounds with a wide range of polarities. The high diffusivity and low viscosity of the super critical mobile phase used in SFC has enabled the rapid resolution of analytes with enhanced chromatographic efficiency compared to other methods [257,259,262]. SFC can be applied to separate highly heterogenous mixtures of complex metabolites, ranging from non-polar to polar in a single chromatographic run, using scCO_2_ with and without co-solvents (e.g., methanol) [263,264,265]. SFC-MS can result in both the interclass and intraclass separation of lipids depending on the chemistry of the stationary phase, as both hydrophilic and hydrophobic columns can be used for separation [67,266,267]. Resolution of isobaric and isomeric species can also be achieved using SFC [266,268,269]. Bamba et al. reported an SFC-MS method that can simultaneously resolve and detect diverse lipid species from 14 classes, including GGLs (MGDG and DGDG) and cerebrosides, in a single run [266]. In the study, lipid classes were resolved using a cyano column, whereas individual molecular species were separated using an octadecylsilyl column, enabling the direct analysis of complex lipids from crude extract. An SFC-MS/MS method based on mixed-mode chromatography was reported by Yamada et al. [269], where the hydrophobic octadecylsilyl column, with an embedded polar functional group, resulted in the separation of polar lipids based on their head group as well as fatty acyl chains. The method improved the resolution between the isomeric molecular species of two different phospholipid classes that usually coelute in RPLC [269]. Similarly, Lísa and Holčapek developed a rapid SFC-MS method that enabled the detection of 436 lipid species from 24 lipid classes within six minutes of analytical runtime [270]. The method enabled the separation of lipids primarily based on the polar head group, similar to HILIC, along with the partial intraclass separation of molecular species.

Despite the advantages of comprehensive lipidomic profiling, the application of SFC-MS to marine glycolipids is still limited. Advancements in the methods and instrumentation of SFC-MS, enabling the rapid resolution as well as comprehensive detection of complex lipids, is promising for lipidomic applications [67,257], and could decrease sample purification requirements [266].

### 4.4. 2D-LC-MS

The 2D-LC-MS method enables the rapid characterisation of polar and non-polar metabolites in a single chromatographic run with enhanced resolution of individual molecular species [70,271,272]. The approach enhances the detection of less abundant lipid species by improving separation and reducing potential ion suppression in the MS. HILIC and RPLC are used in most published 2D-LC-MS-based lipidomic studies [69,70,273]. Other forms of chromatography, such as NPLC, mixed-mode chromatography, anion exchange chromatography, and SFC can also be combined with RPLC to provide a second-dimension separation of lipid subclasses [68,274,275,276,277]. Holčapek et al. developed a continuous 2D-LC-MS system comprising an RPLC and HILIC that enabled the identification of 143 lipid species, including 9 hexosylceramides, using human plasma and porcine brain samples [278]. In their study, RPLC was used in the first dimension followed by HILIC in the second dimension to rapidly resolve co-eluted lipid species.

Although laborious, offline 2D-LC-MS is generally superior in terms of detection capability for diverse lipid species, as each lipid class/subclass fraction that is collected from the first-dimension LC is preconcentrated and reconstituted in an appropriate solvent prior to analysis in second-dimension LC coupled with MS [69,278]. Narváez-Rivas et al. reported more than 800 lipid molecular species using an offline 2D-LC-MS method based on monolithic silica and RPLC columns [276]. As highlighted in the study, mixed-mode retention of monolithic silica was crucial for resolving 22 lipid classes in the first dimension, 13 fractions of which were analysed using second-dimension RPLC-MS. Another offline 2D-LC based on SFC and RPLC-MS was reported by Si et al. for analysing complex gangliosides [68]. In total, 79 compounds belonging to 22 classes of gangliosides were detected using the method, compared to 1D-LC, where only a fourth of molecules were detected. An HILIC-RPLC-based offline 2D-LC-MS method was reported by Sorenson et al., where more than 1000 lipid species were identified from a human plasma sample [69]. The improvement in the peak capacity and the lipidome coverage compared to 1D analysis was attributed to sample preconcentration prior to the second-dimensional analysis.

In all of the examples discussed above, the primary objective of 2D-LC was to enhance the detection of diverse lipid species, including the less abundant ones by improving the resolving power. Despite the benefits of the approach, application of 2D-LC-MS targeted for comprehensive screening of marine glycolipid classes cannot be found in the recent literature. Most 2D-LC-MS/MS studies analysed the total lipids obtained from biphasic lipid extractions, such as Bligh and Dyer, Folch, and MTBE methods [70,271,274,279]. Some polar glycolipids can remain at the interface or in the aqueous phase, limiting recovery. In such cases, an extraction method that includes sequential monophasic extractions to extract glycolipids from a wide polarity range is necessary for a comprehensive analysis of glycolipids using 2D-LC-MS/MS.

Despite 2D-LC being more effective for the enhanced resolution of complex lipids, an optimised 1D separation on certain stationary phases can also improve the separation/detection of glycolipid species. An example of this is the application of a phenyl-hexyl column by Gobburi et al. for the separation and analysis of gangliosides present in mouse brain samples [254]. Baseline resolution of nine major ganglioside species from four subclasses (GQ1b, GT1b, GD1a, and GM1) was reported in the study, which was associated with mixed-mode retention mechanism offered by the phenyl-hexyl stationary phase. Individual molecules consisting of different ceramide, units were resolved within the same retention time window of each subclass, which were separated based on the number of sialylated sugars [254]. The method could potentially remove the need for complex 2D-LC configurations while achieving similar degrees of resolution, but has limitations in that it is only useful for the analysis of glycolipids that vary in the number of charged sugars, and is not applicable to those with neutral polar head groups.

### 4.5. ESI-MS/MS Methods

Optimisation of MS parameters, such as ion source temperature, declustering potential, and the polarity of the ESI interface is important for optimised detection after separation. The temperature and voltage applied at the ion source can be tuned to the desired conditions for maximising the yield of primary precursor ions while reducing multiple adducts. ESI is a soft ionisation technique commonly used in LC-MS. However, a higher interface temperature can sometimes result in significant in-source fragmentation, leading to the false identification of an analyte. This is particularly evident for some glycolipids (e.g., gangliosides), where the cleavage of sialylated sugars transforms one ganglioside into another, leading to incorrect molecular assignment. Interface temperatures above 400 °C tend to be detrimental for gangliosides analysis [253].

ESI polarity also affects the detection sensitivity of glycolipids as the acidic GSLs (e.g., gangliosides), and acidic GGLs (e.g., SQDG) are preferentially detected in negative ESI mode [47,56], while neutral glycolipids are better detected in positive mode [37,280]. Zhang et al. showed that the intensity and detectability of lipid species is dependent on the lipid headgroup, resuspension solvent, and the mobile phase buffer used in LC-ESI-MS analysis [63]. The study demonstrated that neutral GSLs (galCer) were sufficiently ionised in positive ESI mode when using ammonium formate as a mobile-phase buffer/additive, in contrast to the suppression of ion intensity in negative mode in the presence of an ammonium acetate buffer. As highlighted by Lu et al., the use of both ESI polarities is preferable, as it results in wider lipidome coverage in the LC-MS analysis [280].

A wide of range of MS detectors, such as QTOF-MS [57,63,281,282], triple quadrupole (QQQ)-MS [9,234,283], and the higher-resolution Orbitrap-MS [15,90,122,232,284], are used in LC-MS/MS analysis of marine lipids. QTOF and Orbitrap are used for both targeted and untargeted analyses, assisting in the structural characterisation of both known and unknown molecules. In untargeted analyses, raw LC-MS/MS data consisting of analyte elution order (retention time), precursor (MS), and product ions (MS/MS) spectra are processed using automated annotation software packages, such as MS-DIAL [285], to identify glycolipid molecules. MS-DIAL is an open-source tool commonly used for LC-MS/MS data processing and the annotation of lipid molecules. It has an integrated lipidomics library composed of in silico fragmentation data from LipidBlast [286], as well as experimental MS/MS data for the rule-based annotation of lipid molecules, covering major glycolipid subclasses like MGDG, DGDG, SQDG, and hexosylceramides [285,286,287]. The use of MS/MS spectral libraries from different databases, such as MassBank of North America (MoNA) [288] and Global Natural Products Social Molecular Networking (GNPS) [289], can enhance the number of annotations of lipid molecular species [72]. Several other lipid annotation software packages, such as LipidMatch [290], or vendor-specific tools like LipidAnnotator (Agilent Technologies, Santa Cara, CA, USA) [291] and LipidSearch (Thermo Fisher Scientific, Waltham, WA, USA), are used in lipidomics [71]. Each software has its own limitations around annotation rules based on pre-defined fragmentation patterns generated from specific analytical instruments/conditions, which may lead to either false-positive or limited identifications [292,293]. Exporting alignment results from one software and verifying them using another software/annotation workflow for consensus across different data analysis platforms, and even manual curation of data, can enhance the confidence of identification [292,294]. Advanced software like LipidIn [295] has recently been released that uses fragmentation hierarchical library and machine learning for drastically reducing false-positive annotations. The glycolipid-specific annotation tool DANGO [296] has recently been released for the identification of permethylated GSLs using the GRITS [297] platform, which was originally developed for glycomics data processing. This tool combines both the glycan and lipid databases to generate simulated fragment structures for comparison with experimental data for annotation. Manual review of specific fragments is essential to filter those that are relevant to the experimental data and reduce false-positive annotations [296]. With advancements in bioinformatics and machine learning, mass spectrometric data analysis workflow is improving for rapid and comprehensive characterisations of lipids, including glycolipid subclasses. In-depth analyses of individual software packages are beyond the scope of this review. The advantages and limitations of software packages used for automated annotation of lipids are discussed in recent reviews [71,298,299].

QQQ-MS is applied for the targeted analysis of compounds using the precursor ion scan and neutral loss scan modes in MS/MS, which enables the rapid identification of molecules sharing common diagnostic fragments. For example, for GGLs, SQDG molecules can be detected by selecting a product ion of *m*/*z* 225 in negative ESI mode, while MGDGs and DGDGs can be detected by selecting a neutral loss scan of *m*/*z* 197 and 359, respectively, in positive ESI mode [47,234]. The MRM approach based on MS/MS can be applied for quantifying target molecules with established fragmentation data. In order to obtain reliable and reproducible MRM peak data from selected precursor–product transitions of each molecule, it is necessary to optimise the collision-induced dissociation conditions, such as collision energy and declustering potential, in MS/MS. Glycolipid subclasses such as GGLs (MGDG, DGDG, and SQDG) can be quantified using the MRM method by targeting their characteristic product ions [47,123].

Lipidomics and glycolipidomics studies have benefitted from the recent application of ion mobility (IM)-MS, which provides additional resolving power for the separation and detection of lipids isomers [49,175,300,301]. The shape, size, and molecular conformation of charged precursor ions affect the mobility that is detectable by the MS detector. The differential mobility is measured by the collision cross-section value, which is unique to the chemical structure and three-dimensional conformation of individual molecules. This can enhance the confidence of identification, specifically among isomeric and isobaric species that are difficult to distinguish using *m*/*z* values, retention time, and tandem mass spectra alone [300,302,303]. Advances in IM-MS technologies, which provide ultra-high-resolution capabilities, were recently reviewed by Naylor and Nagy [304]. Application of IM-MS using structures for lossless ion manipulations (SLIM) has been reported by Wojcik et al., which enabled the resolution of ganglioside isomer GD1a and GD1b structures [305]. The SLIM technique has also been applied by Moser et al., where two additional isomeric forms of GD1 (GD1α and GD1c) and four isoforms of GT (GT1a, GT1b, GT1α, and GT1c) were resolved from a lipid extract from a mouse brain without the need for prior LC separation [306]. Recently, another high-resolution IM-MS (cyclic ion mobility separation MS) technique was reported by Naylor and Nagy, where glycolipid isomers from the cerebroside subclass (α- and β-galCer) were effectively separated when analyte adducts were formed with lithium or deprotonated [307]. The study highlights the usefulness of post column permethylation or metal adduction for the enhanced resolution of lipid isomers in IM-MS. IM-MS is a particularly promising method for the analysis of structurally similar and less abundant glycolipids.

Advances in LC-MS/MS have revolutionised the way in which complex lipids are analysed, enabling the rapid molecular characterisation of individual lipids in complex mixtures. The mode of separation, chromatographic features, mass spectrometer sensitivity, and resolving power vary greatly depending on the type of glycolipid subclass, purity level, and sample source. LC-MS/MS has some limitations, including its inability to distinguish between some isomers, such as the position of unsaturation in the lipidic chain. Additional techniques, such as IM-MS and online ozonolysis, can overcome these limitations [307,308].

## 5. Conclusions

Marine glycolipids have been shown to be useful in the development of biosurfactants, emulsifiers, and ingredients for cosmetics, nutraceuticals, and pharmaceuticals. This is partly due to their physicochemical properties, but also due to their range of biological activities, including anti-inflammatory, antiadipogenic, and antimicrobial activities. Some marine glycolipids have useful functional properties, such as improving skin hydration and epidermal barrier function. Studies targeting glycolipids are mostly limited to microalgae, seaweeds, and some marine invertebrates, such as sea urchin, starfish, and sea cucumber. Marine glycolipids from sources such as fish and shellfish have not been comprehensively analysed due to challenges with extraction, their inability to detect low-abundance species, and the general laboriousness of sample preparation and analysis. Despite the opportunities for new bioproducts, glycolipids remain relatively unexplored compared to other classes of marine lipid.

Glycolipid extracts are prepared primarily using conventional lipid extraction solvents. There are a limited number of studies investigating the efficiency of extraction solvents and techniques targeted to specific glycolipids. Lipidomic approaches are mostly untargeted, and focus on comparisons of total lipid profiles based on molecular diversity and relative abundance. Direct comparisons of glycolipid enrichment efficiency are difficult to perform across current studies due to a lack of a universal quantification approach for individual subclasses. Alternative solvents used for lipid extraction, such as BUME and MTBE, as well as advanced greener extraction techniques like PLE and SFE, are worth examining for glycolipid enrichment. Since SFE are generally effective for removing neutral lipids from the biomass, integrating sequential extraction steps on the residual biomass could potentially simplify glycolipid enrichment without complicated purification procedures. The selection of purification methods for glycolipids depends largely on the extract’s compositional characteristics and the specific subclass targeted. In biomasses that contain a high concentration of ionisable phospholipids, the application of TiO_2_ and ZrO_2_ stationary phases has been instrumental in the purification of GSLs prior to analysis.

Classical TLC-staining methods remain important for glycolipid detection. Coupling TLC with FID using Iatroscan^TM^ has provided a low-cost alternative to separate and quantify lipid classes/subclasses in crude extracts. Unlike the routine TLC-FID method used for lipid class analysis, multistage sequential TLC-partial scan FID has been important in resolving and quantifying subclasses of GGLs (MGDG, DGDG, and SQDG) directly from crude extract. Despite its efficacy, the method remains underutilised for marine glycolipids. Methodological advancements in TLC-MS techniques have enabled the rapid characterisation of polar lipids that are less accessible to standard LC-MS methods. TLC-MS could simplify the analysis of marine glycolipid subclasses, and is complimentary to LC-MS/MS.

A range of LC-MS/MS methodologies have been applied to the analysis of glycolipids from marine sources. The structural diversity within the glycolipid class has resulted in the non-uniformity of analytical techniques. Chromatographic methods that are optimised for each glycolipid type/subclass are critical for sensitive detection, comprehensive characterisation, and comparisons between studies. HILIC-MS/MS has been shown to be useful for analysing gangliosides subclasses. The resolution of charged/anionic glycolipids using HILIC could be improved by using column chemistries like silica hydride, which minimise unnecessary ionic interaction with analytes. The regioisomeric resolution of glycolipid structures using HILIC can be applied to structure elucidation within each subclass; for instance, between GD1a and GD1b, or between gluCer and galCer. The targeted analysis of purified subclass fractions, such as gluCer and SQDG, using RPLC-MS/MS enable characterisations at the molecular species level. Due to a lack of marine-specific glycolipid standards, glycolipids are often semi-quantified either as the sum of molecular species belonging to each subclass, or are expressed as relative peak area to an internal standard in LC-MS. Quantitative analysis could be improved by using synthetic marine-like glycolipids, along with isotopically labelled internal standards in targeted studies.

Total lipidomic investigations have enabled the rapid identification of some glycolipids from crude extracts. Improvements in chromatography, mass spectrometric detection, and data processing are important for improved LC-MS/MS method development. The resolving power of SFC and 2D-LC could be utilised for improving marine glycolipid detection. Coupling LC with higher-resolution MS instruments such as IM-MS is important for differentiating certain structural isomers. While numerous software packages are available to support the automated annotation of glycolipid molecular species in lipidomic workflow, building a marine-specific MS/MS structural database of glycolipids is crucial to enhance the number of annotations of unique GGLs and GSLs.

Overall advancements in TLC and LC-MS/MS techniques have enabled the rapid identification and semi-quantification of glycolipid subclasses and individual molecules. With improved chromatography and data analysis, it is possible to characterise marine glycolipids in complex mixtures. As new bioactivities and functional properties of marine glycolipids are discovered, these new rapid analysis methods can enable the rapid characterisation of glycolipids in complex lipid extracts, facilitating the development of new and novel bioproducts.

## Figures and Tables

**Table 1:** Biphasic solvent extraction methods used in the glycolipid analytical workflow.

Biphasic Solvent Systems	Frequency of Application in Glycolipids Analysis	Advantages	Disadvantages	Application Notes
Chloroform:methanol based Bligh and Dyer and Folch methods [80,81]	Frequent use	Wide coverage of total lipidome in organic phase.	Solvent toxicity and laborious steps involved in phase partitioning. Less selective for glycolipids; co-extraction of pigments from marine samples. Potential losses of glycolipids in interface emulsion layer. Aqueous phase needs to be recovered for gangliosides.	Method may require modifications based on sample composition for effective phase separation. Further purifications are often essential.
Butanol:methanol based BUME method [85]	Limited use	Rapid extraction in upper organic phase. Lipids recovery similar to chloroform:methanol.	Lack of in-depth studies targeting marine glycolipids.	Good enrichment of polar lipids in non-marine samples. More investigations are essential.
Methyl-tert-butyl-ether:methanol based Matyash method [84]	Limited use	Rapid extraction in upper organic phase. Lipid recovery similar to chloroform:methanol.	Limited information on glycolipids enrichment.	More studies are essential, given the lower polarity of the solvent system.

**Table 2:** Advanced lipid extraction techniques relevant to glycolipid enrichment in lipid extracts.

Advanced Lipid Extraction Methods	Sample Types	Targeted Glycolipid Type/Subclass	Enrichment Levels Compared to Conventional Methods	Analytical Approach	Application Notes
Pressurised fluid extraction (PLE) using hexane:isopropanol	Marine microalgae	Total glycolipids	Higher	NPLC-ELSD-based quantitation [96]	More investigations targeting other subclasses of glycolipids from a range of marine biomasses other than microalgae are essential
Ultrasonic assisted extraction (UAE) using hexane:isopropanol	Marine microalgae	Total glycolipids	Higher	NPLC-ELSD-based quantitation [96]	
UAE using ethanol		Total lipids, including GGLs (MGDG, DGDG)	Higher	LC-MS/MS peak area analysis using class-specific internal standards [98]	
UAE using ethanol		Polar lipids, including DGDG and SQDG	Comparable profile	Molecular diversity and relative abundance analysis using LC-MS/MS [16]	
Supercritical fluid extraction (SFE) (scCo_2_ + ethanol as co-solvent)	Marine microalgae	Polar lipids, including total glycolipids	Lower	Gravimetry of fractions, as well as quantitation based on LC-MS peak area/mass of extract [93]	SFE and DME, including sequential extraction steps, can be examined to recover glycolipids from residual biomass
Dimethyl ether extraction (DME)	Non-marine biomass	Total fatty acids associated with neutral and polar lipids, including GGLs (MGDG, DGDG, SQDG) and cerebrosides	Lower	Analysis of fatty acids from neutral lipids and polar lipids/glycolipids, along with qualitative TLC tests on biomass residues [110]	

ELSD: Evaporative light-scattering detector.

**Table 3:** TLC techniques used for detection of various glycolipid subclasses.

Samples	Glycolipid Subclasses Resolved	TLC Approach	TLC Solvent Systems	Detection Method/Staining	Ref.
Purified glycolipid fractions	MGDG, DGDG, and SQDG	Single development TLC	Chloroform:methanol:water	Orcinol-sulfuric acid	[60,73,75,148]
	Neutral GSLs (cerebroside subclasses)				[128,136,145,149]
	Acidic GSLs (Ganglioside subclasses)		Chloroform:methanol:water with added salt (e.g., 0.2% CaCl_2_)	Resorcinol–hydrochloric acid	[27,128,142,145,150]
	Gangliosides, including minor subclasses such as O-acetylated and lactonized gangliosides	2D-TLC			[134,151,156,157]
Crude lipids	MGDG, DGDG, and SQDG	Multiple/sequential development TLC	Multiple solvent systems of different polarity	TLC coupled with partial-scan FID	[58,60]

Ref.: References.

**Table 4:** TLC coupled methods applicable for detection and quantitation of marine glycolipids.

	TLC Coupled Techniques for Glycolipids Analysis		
	TLC-Densitometry	TLC-FID	TLC-MS
Glycolipids identification level	Class/subclass level; requires staining reagent/standards.	Class/subclass level; requires standards.	Subclass and molecular species level.
Quantification	Time-consuming due to the requirement of derivatization.	Rapid and robust; allows for direct quantification without derivatization.	Allows for relative quantitation, but prone to ionisation sensitivity (require standards).
Advantages	Low-cost, widely used and well-established classical method. Wide range of TLC stationary phases available for better retention and selectivity.	Rapid analysis and low-cost instrumentation.	Higher sensitivity to low-abundance glycolipid species.
Disadvantages	Requires staining/derivatization. Limited application due to a lack of marine specific standards for glycolipids.	Resolving power can be lower due to the limited choice of chromarod stationary phases (silica and alumina).	Expensive instrumentation for developing a robust TLC-MS interface.
Application notes	Resolving power can be improved by automated gradient development, 2D-TLC, and use of modified-phase TLC plates.	Special approaches like multiple-sequential-development TLC and partial-scan-FID can improve resolution for glycolipids.	Advanced elution-based interfaces allow for the direct structural characterisation of glycolipids with enhanced resolution and sensitivity.
References	[142,160,166,169]	[58,60,179,210,211]	[61,185]

**Table 5:** Summary of recent LC-MS/MS analyses of marine extracts reporting the molecular diversity and/or abundance of glycolipid structures.

Sample Source	Extraction and Enrichment Method	LC-MS/MS Method	Types and Number of Glycolipid Molecular Species Detected	Quantification Method	References
Brown alga wakame (*U. pinnatifida*)	Modified Bligh and Dyer method. Sulfolipids enrichment by normal-phase SPE, eluted with acetonitrile:water (80:20, *v*/*v*) and 0.1% formic acid.	LC coupled with Q-Exactive Quadrupole-Orbitrap-MS. Bare silica HILIC for total lipids analysis. C18 RPLC for sulfolipids analysis.	16 SQDG, 6 SQMG, 12 DGDG, and 5 DGMG among >200 polar lipid species.	Determination of relative abundance of molecular species without quantification.	[15]
Three brown seaweeds (*L. japonica*, *U. pinnatifida*, and *S. natans*)	Modified Bligh and Dyer method.	C18 RPLC coupled with QTOF-MS.	30–66 MGDG, 20–45 DGDG, 13–48 SQDG, 3–4 MGMG, 19–121 acylated MGDG, 2–4 DGMG, 6–35 acylated DGDG, 1 SQMG, and 1–9 hexosylceramides among 675 lipid molecules across three samples.	Lipid class-specific semi-quantification using an external standard method.	[14]
Four shellfish species (*R. philippinarum*, *O. gigas*, *C. farreri* and *M. edulis*)	Modified Folch method.	C18 RPLC coupled with Triple TOF-MS.	4–16 hexosylceramides among >600 molecules across four samples.	Lipid class-specific semi-quantification using an internal standard method.	[228]
Eight echinoderm species (*A. japonicus*, *A. molpadioides*, *C. frondosa*, *P. californicus*, *T. ananas*, *A. amurensis*, *G. crenularis*, and *S. nudus*)	Modified Folch method.	C18 RPLC coupled with Triple TOF-MS.	4–8 SQDG and 39–73 hexosylceramides among 961 lipid molecules across eight samples.	Lipid class specific semi-quantification using an internal standard method.	[229]
Four fish species (*S. niphonius*, *S. maximus*, *O. keta* and *C. idellus*)	Modified Folch method.	C18 RPLC coupled with QTOF-MS.	Six hexosylceramides detected in *S. maximus* among >700 lipid molecular species across four samples.	Class specific semi-quantification using an external standard method.	[230]
Five marine fish species (*L. campechanus*, *E. lanceolatus*, *S. canaliculatus*, *L. calcarifer* and *K. pelamis*)	*n*-Hexane.	C18 RPLC coupled with Orbitrap-MS.	1–14 hexosylceramides 0–1 MGDG and 0–1 SQDG among >1000 lipid molecules across five samples (detected in positive ESI mode). 3–13 MGDG among 100–300 lipid species (detected in negative ESI mode).	Lipidomic assessment without quantification.	[231]
Marine microalgae (*N. oceanica*)	Monophasic solvent extractions followed by phase partition using the Folch method.	Bare silica HILIC coupled with Q-Exactive-Orbitrap-MS.	15 MGDG, 5 MGMG, 14 DGDG, 2 DGMG, 10 SQDG, and 1 SQMG among 128 lipid species.	Comparison of extraction efficiency based on relative abundance without quantification.	[16]
Marine plankton (*E. huxleyi*)	Modified Folch method.	Bare silica HILIC coupled with Q-Exactive-Orbitrap-MS.	17 MGDG, 8 MGMG, 7 DGDG, 1 DGMG, 24 SQDG, 3 SQMG, and 2 sialylated GSLs composed of KDN among 134 lipid species.	Estimation of total glycolipids in crude extracts using the orcinol colorimetric method. Relative abundance of molecular species without quantification.	[140]
Brown alga (*F. vesiculosus*)	Modified Bligh and Dyer method.	Bare silica HILIC coupled with Q-Exactive-Orbitrap-MS.	26 MGDG, 4 MGMG 27 DGDG, 5 DGMG, 13 SQDG, and 6 SQMG among 187 lipid species.	Relative abundance of molecular species without quantification.	[232]
Six seaweeds samples Chlorophyta (*U. rigida*, *C. tomentosum*) Rhodophyta (*P. palmata*, *G. gracilis*, *P. dioica*), and Ochrophyta (*F. vesiculosus*)	Modified Bligh and Dyer method.	Bare silica HILIC coupled with Q-Exactive-Orbitrap-MS.	6–24 MGDG, 8–12 MGMG, 10–22 DGDG, 3–7 DGMG, 17–26 SQDG, and 1–5 SQMG among 144–275 lipid species across six samples.	Relative abundance of molecular species without quantification.	[8]
Atlantic red seaweed (*G. turuturu*)	Modified Bligh and Dyer method.	Bare silica HILIC coupled with Q-Exactive-Orbitrap-MS.	16 MGDG, 13 DGDG, 13 MGMG, 4 DGMG, 20 SQDG, and 8 SQMG.	Relative percentage of molecular species based on the normalised peak area of each extracted ion chromatogram.	[53]
Green seaweed (*U. rigida*)	Modified Bligh and Dyer method.	Bare silica HILIC coupled with Q-Exactive-Orbitrap-MS.	9 MGDG, 13 DGDG, 20 SQDG, 6 MGMG, 6 DGMG, and 5 SQMG among 150 lipid species.	Relative quantification at subclass level by the integration of molecular peak areas. Relative abundance measurement based on the normalised peak area of each extracted ion chromatogram relative to the internal standard for the same class.	[233]
Green macroalga (*U. rigida*)	Modified Bligh and Dyer method followed by normal-phase SPE for polar lipids fraction. Glycolipids eluted with acetone:methanol (9:1, *v*/*v*).	Bare silica HILIC coupled with Q-Exactive-Orbitrap-MS.	27 MGDG, 13 MGMG, 13 DGDG, 9 DGMG 20 SQDG, and 5 SQMG among 202 polar lipid species.	Polar lipid profiling without quantification.	[120]
Sugar kelp (*S. latissimi*)	Modified Bligh and Dyer method followed by normal-phase SPE for polar lipids fraction. Glycolipids eluted with acetone:methanol (9:1, *v*/*v*).	Bare silica HILIC coupled with Q-Exactive-Orbitrap-MS.	14 MGDG, 15 DGDG, 25 SQDG, and 3 SQMG among 197 lipid molecules.	Polar lipid profiling without quantification.	[52]
Sacoglossan sea slug (*E. viridis*), and Green alga (*C. tomentosum*)	Modified Bligh and Dyer method followed by normal-phase SPE for polar lipids fraction. Glycolipids eluted with acetone:methanol (9:1, *v*/*v*).	Bare silica HILIC coupled with Q-Exactive-Orbitrap-MS.	2 MGDG, 6 DGDG, and 12 SQDG among 62 molecular species from *E. viridis*. 2 MGDG, 6 DGDG, and 16 SQDG among 76 molecular species *C. tomentosum*.	Polar lipid profiling without quantification.	[122]
Red seaweed (*C. crispus*)	MTBE method.	Bare silica HILIC coupled with Linear Ion Trap-MS.	30 SQDG, 19 DGDG, and 4 galactosylceramides.	Polar lipid profiling without quantification.	[64]
Green seaweed (*C. tomentosum*)	Methanol extraction followed using TLC, and re-extraction of polar lipids using chloroform:methanol (2:1, *v*/*v*).	Bare silica HILIC coupled with Linear Ion Trap-MS.	7 SQDG, 4 SQMG, 13 DGDG, and 10 MGDG.	Polar lipid profiling without quantification.	[65]
Red alga (*Gracilaria* spp.)	Modified Bligh and Dyer method followed by normal-phase SPE for polar lipids fraction. Glycolipids eluted with acetone:methanol (9:1, *v*/*v*).	Bare silica HILIC coupled with Linear Ion Trap-MS for total lipids analysis. Direct infusion ESI-QTOF-MS/MS for fractions analysis.	9 MGDG, 10 DGDG, 12 SQDG, and 3 SQMG among 147 lipid molecular species.	Polar lipid profiling without quantification.	[121]
Marine plankton	Modified Bligh and Dyer method.	NPLC coupled with QQQ-MS.	MGDG, DGDG, and SQDG among 9 polar lipid subclasses.	Sum quantification of molecular species of each subclass based on characteristic neutral loss scan for each subclass.	[234]
Microalgae species (*C. cryptica*, *N. salina*, *T. weissflogii*, *A. minutum* and *A. tamutum*)	MTBE method followed by silica gel column chromatography. Glycolipids eluted with acetone:methanol (9:1, *v*/*v*). Additional purification of GGLs (MGDG, DGDG, and SQDG) using Sephadex LH-20.	Biphenyl RPLC coupled with Q-Exactive-Hybrid Quadrupole-Orbitrap-MS.	At least 21 MGDG, 14 DGDG, 7 SQDG among >350 lipid species across five samples.	Molecular diversity and relative abundance of lipid molecular species. Absolute quantification of lipid species using five standards representing the lipid classes, including MGDG and SQDG.	[90]
Sea cucumber species (*A. molpadioides*, *C. frondosa*, and *A. japonicus*)	Sequential extractions using chloroform:methanol (2:1, *v*/*v*), followed by re-extraction with ethyl acetate:n-butanol (2:1, *v*/*v*), and precipitation of residue with acetone. Glucocerebroside enrichment by normal phase silica gel column chromatography, eluted with chloroform:methanol (25:1 to 10:1, *v*/*v*).	C18 RPLC coupled with Ion Trap-MS.	12, 26, and 52 glucocerebroside molecular species across three samples. Characteristic glucocerebrosides consisting of C17/C18 sphingoid bases (d17:1 and d18:2) and C18-C25 saturated/monounsaturated and hydroxy/non-hydroxy fatty acid groups.	Structural characterisation without quantification.	[235]
Starfish (*A. amurensis*)	Bligh and Dyer method. Glucocerebroside enrichment using normal-phase silica gel column chromatography, eluted with chloroform:methanol:water (90:10:0.1, *v*/*v*/*v*).	C18 RPLC coupled with Ion Trap-TOF-MS.	>23 molecular peaks of cerebrosides including several structural isomers. Three groups of cerebrosides: one consisting of C22 LCBs and <C16 fatty acyl group, and the other two consisting of C18 LCBs and either >C23 or <C18 fatty acyl groups with various number and position of unsaturation(s). Key structures consisting of trihydroxy and branched triene sphingoid bases.	Structural characterisation without quantification.	[26]
Sea cucumber (*P. graeffei*)	Chloroform:methanol (2:1, *v*/*v*) followed by mild saponification (potassium hydroxide in methanol), phase partition and recovery of chloroform phase. Glucocerebrosides enrichment by normal-phase SPE, eluted with chloroform:methanol (100:0 to 90:10, *v*/*v*).	C18 RPLC coupled with QTOF-MS.	89 cerebroside molecular species. Unique structures possessing d17:1 and branched sphingoid bases and those with hydroxy fatty acids.	Structural characterisation without quantitation.	[57]
Sea cucumber (*B. marmorata*, *I. fuscus*, *H. polli*, *H. mexicana*, *C. frondosa* and *P. califormicus*)	Svennerholm and Fredman method. Gangliosides purified by SPE using C8 cartridges.	Amino HILIC coupled with Q-Exactive Orbitrap-MS.	17 subclasses of GM, GD, GT, GQ, and GP gangliosides, consisting of different substituents in sialic head (sulphate, fucose and inositolphosphate).	Semi-quantitative analysis using GD3 isolated from bovine milk as an external standard.	[30]
Sea urchin (*S. nudus*, *H. pulcherrimus*, and *G. crenularis*)	Svennerholm and Fredman method. Gangliosides purified by SPE using C8 cartridges.	Amino HILIC coupled with Q-Exactive-Orbitrap-MS.	14 subclasses of gangliosides with three distinct sialic acid features (Neu5Ac, Neu5Gc, and KDN), along with sulphated sialic acids.	Semi-quantitative analysis using GM1, GD1 and GT1 as external standards.	[31]
Sea urchin (*S. nudus*)	Svennerholm and Fredman method. Gangliosides purified by C8 column, followed by anion exchange chromatography using DEAE-Sephadex A25.	C8 RPLC coupled with QQQ-MS.	>50 molecules of GM gangliosides. Structures composed of two distinct sialic acid types, Neu5Ac and Neu5Gc, and their sulphated forms.	Ganglioside profiling based on MS/MS- and MRM-based relative quantification.	[236]
Fish fillets (*C. nudipinnis*, *R. faughni*, *O. tshawytscha*, *C. auratus*) along with other non-marine food samples	Modified Svennerholm and Fredman method. Gangliosides enriched by SPE using C18 cartridges.	Amino-propyl HILIC coupled with Orbitrap-MS.	Five subclasses of gangliosides, including GM3, GD3, GD1a, GD1b, GT1b, and GM2.	Subclass levels quantification of gangliosides using both ab external standard and standard addition technique.	[94]

## Abbreviations

The following abbreviations are used in this manuscript:

2D-LC	Two-dimensional liquid chromatography
9-AA	9-amino acridine
BUME	Butanol:methanol
DEAE	Diethylaminoethyl
DESI	Desorption electrospray ionisation
DGDG	Digalactosyldiacylglycerol
DGMG	Digalactosylmonoacylglycerol
DHB	Dihydroxy benzoic acid
DME	Dimethyl ether
ESI	Electrospray ionisation
FID	Flame ionisation detection
galCer	Galactosylceramide
GD	Disialo ganglioside
GGL	Glycoglycerolipid
gluCer	Glucosylceramide
GM	Monosialo ganglioside
GQ	Tetrasialo ganglioside
GSL	Glycosphingolipid
GT	Trisialo ganglioside
HILIC	Hydrophilic interaction liquid chromatography
HIP	Hexane:isopropanol
IM-MS	Ion mobility mass spectrometry
KDN	2-keto-3-deoxy-D-glycero-D-galacto-nonulosonic acid
LC	Liquid chromatography
LCB	Long chain base
LESA	Liquid extraction surface analysis
MAG	Monoacylglycerol
MALDI	Matrix assisted laser desorption ionisation
MGDG	Monogalactosyldiacylglycerol
MGMG	Monogalactosylmonoacylglycerol
MRM	Multiple reaction monitoring
MS	Mass spectrometry
MTBE	Methyl-tert-butyl-ether
Neu5Ac	N-acetyl neuraminic acid
Neu5Gc	N-glycolyl neuraminic acid
NPLC	Normal-phase liquid chromatography
PLE	Pressurised liquid extraction
QQQ	Triple quadrupole
QTOF	Quadrupole time of flight
RPLC	Reverse-phase liquid chromatography
scCO2	Supercritical carbon dioxide
SFC	Supercritical fluid chromatography
SFE	Supercritical fluid extraction
SLIM	Structures for lossless ion manipulations
SPE	Solid-phase extraction
SQDG	Sulfoquinovosyldiacylglycerol
SQMG	Sulfoquinovosylmonoacylglycerol
TLC	Thin-layer chromatography
UAE	Ultrasonic-assisted extraction
UV	Ultraviolet

## References

[1] Tabandeh M., Goh E.W., Salman A.A., Heidelberg T., Duali Hussen R.S. (2018). Functionalized glycolipids for potential bioconjugation of vesicles. Carbohydr. Res..

[2] Dusane D.H., Pawar V.S., Nancharaiah Y.V., Venugopalan V.P., Kumar A.R., Zinjarde S.S. (2011). Anti-biofilm potential of a glycolipid surfactant produced by a tropical marine strain of Serratia marcescens. Biofouling.

[3] Anestopoulos I., Kiousi D.-E., Klavaris A., Maijo M., Serpico A., Suarez A., Sanchez G., Salek K., Chasapi S.A., Zompra A.A. (2020). Marine-Derived Surface Active Agents: Health-Promoting Properties and Blue Biotechnology-Based Applications. Biomolecules.

[4] Lourith N., Kanlayavattanakul M. (2009). Natural surfactants used in cosmetics: Glycolipids. Int. J. Cosmet. Sci..

[5] Cheng-Sánchez I., Sarabia F. (2018). Chemistry and Biology of Bioactive Glycolipids of Marine Origin. Mar. Drugs.

[6] Jala R.C.R., Vudhgiri S., Kumar C.G. (2022). A comprehensive review on natural occurrence, synthesis and biological activities of glycolipids. Carbohydr. Res..

[7] Maciel E., Leal M.C., Lillebø A.I., Domingues P., Domingues M.R., Calado R. (2016). Bioprospecting of Marine Macrophytes Using MS-Based Lipidomics as a New Approach. Mar. Drugs.

[8] Lopes D., Melo T., Rey F., Costa E., Moreira A.S.P., Abreu M.H., Domingues P., Lillebø A.I., Calado R., Rosário Domingues M. (2021). Insights of species-specific polar lipidome signatures of seaweeds fostering their valorization in the blue bioeconomy. Algal Res..

[9] Rey F., Melo T., Lopes D., Couto D., Marques F., Domingues M.R. (2022). Applications of lipidomics in marine organisms: Progress, challenges and future perspectives. Mol. Omics.

[10] Fattorusso E., Mangoni A. (1997). Marine Glycolipids. Fortschritte der Chemie Organischer Naturstoffe/Progress in the Chemistry of Organic Natural Products.

[11] Cepas V., Gutiérrez-Del-Río I., López Y., Redondo-Blanco S., Gabasa Y., Iglesias M.J., Soengas R., Fernández-Lorenzo A., López-Ibáñez S., Villar C.J. (2021). Microalgae and Cyanobacteria Strains as Producers of Lipids with Antibacterial and Antibiofilm Activity. Mar. Drugs.

[12] Ishizuka I., Yamakawa T., Wiegandt H. (1985). Glycoglycerolipids. New Comprehensive Biochemistry.

[13] Plouguerné E., da Gama B.A., Pereira R.C., Barreto-Bergter E. (2014). Glycolipids from seaweeds and their potential biotechnological applications. Front. Cell. Infect. Microbiol..

[14] Wang H., Yang L., Wang X., Cong P., Xu J., Xue C. (2022). Comprehensive Lipidomic Analysis of Three Edible Brown Seaweeds Based on Reversed-Phase Liquid Chromatography Coupled with Quadrupole Time-of-Flight Mass Spectrometry. J. Agric. Food Chem..

[15] Coniglio D., Bianco M., Ventura G., Calvano C.D., Losito I., Cataldi T.R.I. (2021). Lipidomics of the Edible Brown Alga Wakame (*Undaria pinnatifida*) by Liquid Chromatography Coupled to Electrospray Ionization and Tandem Mass Spectrometry. Molecules.

[16] Melo T., Figueiredo A.R.P., da Costa E., Couto D., Silva J., Domingues M.R., Domingues P. (2021). Ethanol Extraction of Polar Lipids from *Nannochloropsis oceanica* for Food, Feed, and Biotechnology Applications Evaluated Using Lipidomic Approaches. Mar. Drugs.

[17] Guo S.-S., Wang Z.-G. (2022). Glyceroglycolipids in marine algae: A review of their pharmacological activity. Front. Pharmacol..

[18] Zhang J., Li C., Yu G., Guan H. (2014). Total synthesis and structure-activity relationship of glycoglycerolipids from marine organisms. Mar. Drugs.

[19] Akbari V., Abedi M., Yegdaneh A. (2020). Bioassay-guided isolation of glycolipids from the seaweed *Gracilaria corticata*. Res. Pharm. Sci..

[20] Barnathan G., Couzinet-Mossion A., Wielgosz-Collin G., La Barre S., Kornprobst J.-M. (2014). Glycolipids from Marine Invertebrates. Outstanding Marine Molecules: Chemistry, Biology, Analysis.

[21] Careaga V.P., Maier M.S., Attaur R. (2014). Cerebrosides from Marine Organisms. Studies in Natural Products Chemistry.

[22] Sugawara T., Zaima N., Yamamoto A., Sakai S., Noguchi R., Hirata T. (2006). Isolation of Sphingoid Bases of Sea Cucumber Cerebrosides and Their Cytotoxicity against Human Colon Cancer Cells. Biosci. Biotechnol. Biochem..

[23] Malyarenko T.V., Zakharenko V.M., Kicha A.A., Kuzmich A.S., Malyarenko O.S., Kalinovsky A.I., Popov R.S., Svetashev V.I., Ivanchina N.V. (2022). New Ceramides and Cerebrosides from the Deep-Sea Far Eastern Starfish Ceramaster patagonicus. Mar. Drugs.

[24] Liu X., Xu J., Xue Y., Gao Z., Li Z., Leng K., Wang J., Xue C., Wang Y. (2015). Sea cucumber cerebrosides and long-chain bases from *Acaudina molpadioides* protect against high fat diet-induced metabolic disorders in mice. Food Funct..

[25] Duan J., Ishida M., Aida K., Tsuduki T., Zhang J., Manabe Y., Hirata T., Sugawara T. (2016). Dietary Cerebroside from Sea Cucumber (*Stichopus japonicus*): Absorption and Effects on Skin Barrier and Cecal Short-Chain Fatty Acids. J. Agric. Food Chem..

[26] Yamaguchi R., Kanie Y., Kanie O., Shimizu Y. (2019). A unique structural distribution pattern discovered for the cerebrosides from starfish *Asterias amurensis*. Carbohydr. Res..

[27] Masson E.A., Sibille E., Martine L., Chaux-Picquet F., Bretillon L., Berdeaux O. (2015). Apprehending ganglioside diversity: A comprehensive methodological approach. J. Lipid Res..

[28] Yu R., Yanagisawa M., Ariga T. (2007). Glycosphingolipid structures. Introduction to Glycoscience; Synthesis of Carbohydrates.

[29] Guo Z. (2022). The Structural Diversity of Natural Glycosphingolipids (GSLs). J. Carbohydr. Chem..

[30] Wang X., Wang X., Cong P., Zhang X., Zhang H., Xue C., Xu J. (2021). Characterizing gangliosides in six sea cucumber species by HILIC-ESI-MS/MS. Food Chem..

[31] Ma Y., Wang X., Wang Z., Cong P., Xu J., Xue C. (2021). Characterization of Gangliosides in Three Sea Urchin Species by HILIC–ESI-MS/MS. J. Agric. Food Chem..

[32] Malyarenko T.V., Kicha A.A., Stonik V.A., Ivanchina N.V. (2021). Sphingolipids of Asteroidea and Holothuroidea: Structures and Biological Activities. Mar. Drugs.

[33] Colombo D., Compostella F., Ronchetti F., Scala A., Toma L., Tokuda H., Nishino H. (2000). Glycoglycerolipid analogues active as anti-tumor-promoters: The influence of the anomeric configuration. Eur. J. Med. Chem..

[34] Morrison I.M. (2006). Glycolipid Analysis. Encyclopedia of Analytical Chemistry.

[35] Christie W.W., Han X., Christie W.W., Han X. (2012). Chromatographic analysis of molecular species of intact phospholipids and glycolipids. Lipid Analysis.

[36] Imbs A.B., Ermolenko E.V., Grigorchuk V.P., Sikorskaya T.V., Velansky P.V. (2021). Current Progress in Lipidomics of Marine Invertebrates. Mar. Drugs.

[37] Cajka T., Fiehn O. (2014). Comprehensive analysis of lipids in biological systems by liquid chromatography-mass spectrometry. Trends Anal. Chem..

[38] Saini R.K., Prasad P., Shang X., Keum Y.S. (2021). Advances in Lipid Extraction Methods-A Review. Int. J. Mol. Sci..

[39] Khot M., Raut G., Ghosh D., Alarcón-Vivero M., Contreras D., Ravikumar A. (2020). Lipid recovery from oleaginous yeasts: Perspectives and challenges for industrial applications. Fuel.

[40] Otero P., Carpena M., Garcia-Oliveira P., Echave J., Soria-Lopez A., Garcia-Perez P., Fraga-Corral M., Cao H., Nie S., Xiao J. (2023). Seaweed polysaccharides: Emerging extraction technologies, chemical modifications and bioactive properties. Crit. Rev. Food Sci. Nutr..

[41] Liu Z.-Y., Zhou D.-Y., Wu Z.-X., Yin F.-W., Zhao Q., Xie H.-K., Zhang J.-R., Qin L., Shahidi F. (2018). Extraction and detailed characterization of phospholipid-enriched oils from six species of edible clams. Food Chem..

[42] Morel J., Catchpole O., Moreno T., Lagutin K., MacKenzie A., Fenton T., Williams A.M. (2024). Extraction of neutral lipids and phospholipids from marine biomasses using subcritical and supercritical fluids. J. Supercrit. Fluids.

[43] Yang K., Han X. (2011). Accurate Quantification of Lipid Species by Electrospray Ionization Mass Spectrometry—Meets a Key Challenge in Lipidomics. Metabolites.

[44] Huang Z., Wu Q., Lu H., Wang Y., Zhang Z. (2020). Separation of Glycolipids/Sphingolipids from Glycerophospholipids on TiO_2_ Coating in Aprotic Solvent for Rapid Comprehensive Lipidomic Analysis with Liquid Microjunction Surface Sampling-Mass Spectrometry. Anal. Chem..

[45] Khoury S., Masson E., Sibille E., Cabaret S., Berdeaux O. (2020). Rapid sample preparation for ganglioside analysis by liquid chromatography mass spectrometry. J. Chromatogr. B.

[46] Antonelli M., Benedetti B., Cavaliere C., Cerrato A., La Barbera G., Montone C.M., Piovesana S., Laganà A. (2019). Enrichment procedure based on graphitized carbon black and liquid chromatography-high resolution mass spectrometry for elucidating sulfolipids composition of microalgae. Talanta.

[47] Fischer J., Treblin M., Sitz T., Rohn S. (2021). Development of a targeted HPLC-ESI-QqQ-MS/MS method for the quantification of sulfolipids from a cyanobacterium, selected leafy vegetables, and a microalgae species. Anal. Bioanal. Chem..

[48] Anesi A., Guella G. (2015). A fast liquid chromatography-mass Spectrometry methodology for membrane lipid profiling through hydrophilic interaction liquid chromatography. J. Chromatogr. A.

[49] Sarbu M., Zamfir A.D. (2018). Modern separation techniques coupled to high performance mass spectrometry for glycolipid analysis. Electrophoresis.

[50] Jiang P., Lucy C.A. (2016). Coupling normal phase liquid chromatography with electrospray ionization mass spectrometry: Strategies and applications. Anal. Methods.

[51] Rustam Y.H., Reid G.E. (2018). Analytical Challenges and Recent Advances in Mass Spectrometry Based Lipidomics. Anal. Chem..

[52] Rey F., Lopes D., Maciel E., Monteiro J., Skjermo J., Funderud J., Raposo D., Domingues P., Calado R., Domingues M.R. (2019). Polar lipid profile of Saccharina latissima, a functional food from the sea. Algal Res..

[53] da Costa E., Melo T., Reis M., Domingues P., Calado R., Abreu M.H., Domingues M.R. (2021). Polar Lipids Composition, Antioxidant and Anti-Inflammatory Activities of the Atlantic Red Seaweed Grateloupia turuturu. Mar. Drugs.

[54] Okazaki Y., Kamide Y., Hirai M.Y., Saito K. (2013). Plant lipidomics based on hydrophilic interaction chromatography coupled to ion trap time-of-flight mass spectrometry. Metabolomics.

[55] Nakajima K., Akiyama H., Tanaka K., Kohyama-Koganeya A., Greimel P., Hirabayashi Y. (2016). Separation and analysis of mono-glucosylated lipids in brain and skin by hydrophilic interaction chromatography based on carbohydrate and lipid moiety. J. Chromatogr. B.

[56] Hájek R., Jirásko R., Lísa M., Cífková E., Holčapek M. (2017). Hydrophilic Interaction Liquid Chromatography–Mass Spectrometry Characterization of Gangliosides in Biological Samples. Anal. Chem..

[57] Jia Z., Li S., Cong P., Wang Y., Sugawara T., Xue C., Xu J. (2015). High Throughput Analysis of Cerebrosides from the Sea Cucumber *Pearsonothria graeffei* by Liquid Chromatography—Quadrupole-Time-of-Flight Mass Spectrometry. J. Oleo Sci..

[58] Gašparović B., Kazazić S.P., Cvitešić A., Penezić A., Frka S. (2015). Improved separation and analysis of glycolipids by Iatroscan thin-layer chromatography–flame ionization detection. J. Chromatogr. A.

[59] Striby L., Lafont R., Goutx M. (1999). Improvement in the Iatroscan thin-layer chromatographic-flame ionisation detection analysis of marine lipids. Separation and quantitation of monoacylglycerols and diacylglycerols in standards and natural samples. J. Chromatogr. A.

[60] Parrish C.C., Bodennec G., Gentien P. (1996). Determination of glycoglycerolipids by Chromarod thin-layer chromatography with Iatroscan flame ionization detection. J. Chromatogr. A.

[61] Engel K.M., Schiller J., Poole C.F. (2023). Applications of thin-layer chromatography to the analysis of lipids. Instrumental Thin-Layer Chromatography.

[62] Engel K.M., Schiller J. (2021). The value of coupling thin-layer chromatography to mass spectrometry in lipid research—A review. J. Chromatogr. B.

[63] Zhang Y.-Y., Liu Y.-X., Zhou Z., Zhou D.-Y., Du M., Zhu B.-W., Qin L. (2019). Improving Lipidomic Coverage Using UPLC-ESI-Q-TOF-MS for Marine Shellfish by Optimizing the Mobile Phase and Resuspension Solvents. J. Agric. Food Chem..

[64] Melo T., Alves E., Azevedo V., Martins A.S., Neves B., Domingues P., Calado R., Abreu M.H., Domingues M.R. (2015). Lipidomics as a new approach for the bioprospecting of marine macroalgae—Unraveling the polar lipid and fatty acid composition of Chondrus crispus. Algal Res..

[65] da Costa E., Melo T., Moreira A.S.P., Alves E., Domingues P., Calado R., Abreu M.H., Domingues M.R. (2015). Decoding bioactive polar lipid profile of the macroalgae *Codium tomentosum* from a sustainable IMTA system using a lipidomic approach. Algal Res..

[66] Toribio L., Martín M.T., Bernal J. (2024). Supercritical Fluid Chromatography in Bioanalysis–A Review. J. Sep. Sci..

[67] Wolrab D., Peterka O., Chocholoušková M., Holčapek M. (2022). Ultrahigh-performance supercritical fluid chromatography/mass spectrometry in the lipidomic analysis. Trends Anal. Chem..

[68] Si W., Liu Y., Xiao Y., Guo Z., Jin G., Yan J., Shen A., Zhou H., Yang F., Liang X. (2020). An offline two-dimensional supercritical fluid chromatography × reversed phase liquid chromatography tandem quadrupole time-of-flight mass spectrometry system for comprehensive gangliosides profiling in swine brain extract. Talanta.

[69] Sorensen M.J., Miller K.E., Jorgenson J.W., Kennedy R.T. (2023). Two-dimensional liquid chromatography-mass spectrometry for lipidomics using off-line coupling of hydrophilic interaction liquid chromatography with 50 cm long reversed phase capillary columns. J. Chromatogr. A.

[70] Rampler E., Schoeny H., Mitic B.M., El Abiead Y., Schwaiger M., Koellensperger G. (2018). Simultaneous non-polar and polar lipid analysis by on-line combination of HILIC, RP and high resolution MS. Analyst.

[71] Valmori M., Marie V., Fenaille F., Colsch B., Touboul D. (2023). Recent methodological developments in data-dependent analysis and data-independent analysis workflows for exhaustive lipidome coverage. Front. Anal. Sci..

[72] Tsugawa H., Satoh A., Uchino H., Cajka T., Arita M., Arita M. (2019). Mass Spectrometry Data Repository Enhances Novel Metabolite Discoveries with Advances in Computational Metabolomics. Metabolites.

[73] Mattos B.B., Romanos M.T.V., Souza L.M.d., Sassaki G., Barreto-Bergter E. (2011). Glycolipids from macroalgae: Potential biomolecules for marine biotechnology?. Rev. Bras. Farmacogn..

[74] Rod-In W., Monmai C., Shin I.S., You S., Park W.J. (2020). Neutral Lipids, Glycolipids, and Phospholipids, Isolated from Sandfish (*Arctoscopus japonicus*) Eggs, Exhibit Anti-Inflammatory Activity in LPS-Stimulated RAW264.7 Cells through NF-κB and MAPKs Pathways. Mar. Drugs.

[75] Yamashita S., Miyazawa T., Higuchi O., Takekoshi H., Miyazawa T., Kinoshita M. (2022). Characterization of Glycolipids in the Strain Chlorella pyrenoidosa. J. Nutr. Sci. Vitaminol..

[76] Svennerholm L., Fredman P. (1980). A procedure for the quantitative isolation of brain gangliosides. Biochim. Biophys. Acta BBA-Lipids Lipid Metab..

[77] Porter M.J., Zhang G.L., Schnaar R.L. (2021). Ganglioside Extraction, Purification and Profiling. J. Vis. Exp..

[78] Leenders R.G., de Jong J.G., Wevers R.A. (1995). Extraction and purification of gangliosides from plasma and fibroblasts before analysis by thin layer chromatography. Ann. Clin. Biochem..

[79] Saito T., Hakomori S.-i. (1971). Quantitative isolation of total glycosphingolipids from animal cells. J. Lipid Res..

[80] Bligh E.G., Dyer W.J. (1959). A rapid method of total lipid extraction and purification. Can. J. Biochem. Physiol..

[81] Folch J., Lees M., Sloane Stanley G.H. (1957). A simple method for the isolation and purification of total lipids from animal tissues. J. Biol. Chem..

[82] Sanyal D., Venkata Subhash G., Saxena N., Kargupta W., Sapre A., Dasgupta S., Inamuddin, Boddula R., Asiri A.M. (2022). Switchable green solvents for lipids extraction from microalgae. Green Sustainable Process for Chemical and Environmental Engineering and Science.

[83] Archanaa S., Moise S., Suraishkumar G.K. (2012). Chlorophyll interference in microalgal lipid quantification through the Bligh and Dyer method. Biomass Bioenergy.

[84] Matyash V., Liebisch G., Kurzchalia T.V., Shevchenko A., Schwudke D. (2008). Lipid extraction by methyl-tert-butyl ether for high-throughput lipidomics. J. Lipid Res..

[85] Löfgren L., Forsberg G.-B., Ståhlman M. (2016). The BUME method: A new rapid and simple chloroform-free method for total lipid extraction of animal tissue. Sci. Rep..

[86] Hara A., Radin N.S. (1978). Lipid extraction of tissues with a low-toxicity solvent. Anal. Biochem..

[87] Sostare J., Di Guida R., Kirwan J., Chalal K., Palmer E., Dunn W.B., Viant M.R. (2018). Comparison of modified Matyash method to conventional solvent systems for polar metabolite and lipid extractions. Anal. Chim. Acta.

[88] Gorgich M., Mata T.M., Martins A.A., Branco-Vieira M., Caetano N.S. (2020). Comparison of different lipid extraction procedures applied to three microalgal species. Energy Rep..

[89] Nechev J.T., Edvinsen G.K., Eilertsen K.E. (2021). Fatty Acid Composition of the Lipids from Atlantic Salmon-Comparison of Two Extraction Methods without Halogenated Solvents. Foods.

[90] Cutignano A., Luongo E., Nuzzo G., Pagano D., Manzo E., Sardo A., Fontana A. (2016). Profiling of complex lipids in marine microalgae by UHPLC/tandem mass spectrometry. Algal Res..

[91] Reis A., Rudnitskaya A., Blackburn G.J., Mohd Fauzi N., Pitt A.R., Spickett C.M. (2013). A comparison of five lipid extraction solvent systems for lipidomic studies of human LDL. J. Lipid Res..

[92] Ryckebosch E., Bruneel C., Termote-Verhalle R., Muylaert K., Foubert I. (2014). Influence of extraction solvent system on extractability of lipid components from different microalgae species. Algal Res..

[93] Servaes K., Maesen M., Prandi B., Sforza S., Elst K. (2015). Polar Lipid Profile of *Nannochloropsis oculata* Determined Using a Variety of Lipid Extraction Procedures. J. Agric. Food Chem..

[94] Fong B.Y., Ma L., Khor G.L., van der Does Y., Rowan A., McJarrow P., MacGibbon A.K.H. (2016). Ganglioside Composition in Beef, Chicken, Pork, and Fish Determined Using Liquid Chromatography–High-Resolution Mass Spectrometry. J. Agric. Food Chem..

[95] Lydic T.A., Busik J.V., Reid G.E. (2014). A monophasic extraction strategy for the simultaneous lipidome analysis of polar and nonpolar retina lipids. J. Lipid Res..

[96] Señoráns M., Castejón N., Señoráns F.J. (2020). Advanced Extraction of Lipids with DHA from Isochrysis galbana with Enzymatic Pre-Treatment Combined with Pressurized Liquids and Ultrasound Assisted Extractions. Molecules.

[97] Elst K., Maesen M., Jacobs G., Bastiaens L., Voorspoels S., Servaes K. (2018). Supercritical CO_2_ Extraction of Nannochloropsis sp.: A Lipidomic Study on the Influence of Pretreatment on Yield and Composition. Molecules.

[98] Pühringer M., Rampler E., Castejón N. (2024). Unwrapping the (glyco-)lipidome in the microalgae *Microchloropsis gaditana*: Effects of eco-friendly extraction methods. Algal Res..

[99] Garcia-Vaquero M., Sweeney T., O’Doherty J., Rajauria G. (2021). Recent Advances in the Use of Greener Extraction Technologies for the Recovery of Valuable Bioactive Compounds from Algae. Recent Advances in Micro and Macroalgal Processing.

[100] Perez-Vazquez A., Carpena M., Barciela P., Cassani L., Simal-Gandara J., Prieto M.A. (2023). Pressurized Liquid Extraction for the Recovery of Bioactive Compounds from Seaweeds for Food Industry Application: A Review. Antioxidants.

[101] Ballesteros-Vivas D., Ortega-Barbosa J.P., Parada-Alfonso F., Ferreira S.R.S., del Pilar Sánchez-Camargo A., Garcia-Vaquero M., Rajauria G. (2022). Supercritical fluid extraction of lipids, carotenoids, and other compounds from marine sources. Innovative and Emerging Technologies in the Bio-Marine Food Sector.

[102] Chemat F., Rombaut N., Sicaire A.G., Meullemiestre A., Fabiano-Tixier A.S., Abert-Vian M. (2017). Ultrasound assisted extraction of food and natural products. Mechanisms, techniques, combinations, protocols and applications. A review. Ultrason. Sonochem..

[103] Mouahid A., Seengeon K., Martino M., Crampon C., Kramer A., Badens E. (2020). Selective extraction of neutral lipids and pigments from *Nannochloropsis salina* and Nannochloropsis maritima using supercritical CO_2_ extraction: Effects of process parameters and pre-treatment. J. Supercrit. Fluids.

[104] Jamalluddin N.A., Ismail N., Mutalib S.R.A., Sikin A.M. (2022). Sc-CO_2_ extraction of fish and fish by-products in the production of fish oil and enzyme. Bioresour. Bioprocess..

[105] Getachew A.T., Jacobsen C., Sørensen A.-D.M. (2024). Supercritical CO_2_ for efficient extraction of high-quality starfish (*Asterias rubens*) oil. J. Supercrit. Fluids.

[106] Catchpole O., Moreno T., Montañes F., Tallon S. (2018). Perspectives on processing of high value lipids using supercritical fluids. J. Supercrit. Fluids.

[107] Yang X., Li Y., Li Y., Ye D., Yuan L., Sun Y., Han D., Hu Q. (2019). Solid Matrix-Supported Supercritical CO_2_ Enhances Extraction of γ-Linolenic Acid from the Cyanobacterium Arthrospira (*Spirulina*) platensis and Bioactivity Evaluation of the Molecule in Zebrafish. Mar. Drugs.

[108] Ji L., Qiao Z., Zhang X., Cheng X., Wang W., Zhang F., Zhou Y., Yuan Y. (2019). Preparation of Ganglioside GM1 by Supercritical CO_2_ Extraction and Immobilized Sialidase. Molecules.

[109] Xu J., Sugawara T., Zhang T., Koretaro T., Xue C. (2023). The Extraction, Separation Technology, and New Product Development of Functional Lipids from Sea Cucumber. Advances in Sea Cucumber Processing Technology and Product Development.

[110] Nekrasov E.V., Tallon S.J., Vyssotski M.V., Catchpole O.J. (2021). Extraction of lipids from New Zealand fern fronds using near-critical dimethyl ether and dimethyl ether–water–ethanol mixtures. J. Supercrit. Fluids.

[111] Bauer M.C., Kruse A. (2019). The use of dimethyl ether as an organic extraction solvent for biomass applications in future biorefineries: A user-oriented review. Fuel.

[112] Zheng Q., Watanabe M. (2022). Advances in low-temperature extraction of natural resources using liquefied dimethyl ether. Resour. Chem. Mater..

[113] Goto M., Kanda H., Wahyudiono, Machmudah S. (2015). Extraction of carotenoids and lipids from algae by supercritical CO_2_ and subcritical dimethyl ether. J. Supercrit. Fluids.

[114] Bauer M.C., Konnerth P., Kruse A. (2023). Extraction of common microalgae by liquefied dimethyl ether: Influence of species and pretreatment on oil yields and composition. Biomass Convers. Biorefin..

[115] Wang T., Zhu L., Mei L., Kanda H. (2024). Extraction and Separation of Natural Products from Microalgae and Other Natural Sources Using Liquefied Dimethyl Ether, a Green Solvent: A Review. Foods.

[116] Grosso C., Valentão P., Ferreres F., Andrade P.B. (2015). Alternative and Efficient Extraction Methods for Marine-Derived Compounds. Mar. Drugs.

[117] Catchpole O., Ryan J., Zhu Y., Fenton K., Grey J., Vyssotski M., MacKenzie A., Nekrasov E., Mitchell K. (2010). Extraction of lipids from fermentation biomass using near-critical dimethylether. J. Supercrit. Fluids.

[118] Wang Q., Oshita K., Takaoka M. (2021). Effective lipid extraction from undewatered microalgae liquid using subcritical dimethyl ether. Biotechnol. Biofuels.

[119] Heinzelmann S.M., Bale N.J., Hopmans E.C., Damsté J.S.S., Schouten S., Meer M.T.J.v.d. (2014). Critical Assessment of Glyco- and Phospholipid Separation by Using Silica Chromatography. Appl. Environ. Microbiol..

[120] Lopes D., Moreira A.S.P., Rey F., da Costa E., Melo T., Maciel E., Rego A., Abreu M.H., Domingues P., Calado R. (2019). Lipidomic signature of the green macroalgae Ulva rigida farmed in a sustainable integrated multi-trophic aquaculture. J. Appl. Phycol..

[121] da Costa E., Melo T., Moreira A.S., Bernardo C., Helguero L., Ferreira I., Cruz M.T., Rego A.M., Domingues P., Calado R. (2017). Valorization of Lipids from *Gracilaria* sp. through Lipidomics and Decoding of Antiproliferative and Anti-Inflammatory Activity. Mar. Drugs.

[122] Rey F., Costa E.d., Campos A.M., Cartaxana P., Maciel E., Domingues P., Domingues M.R.M., Calado R., Cruz S. (2017). Kleptoplasty does not promote major shifts in the lipidome of macroalgal chloroplasts sequestered by the sacoglossan sea slug *Elysia viridis*. Sci. Rep..

[123] Körber T.T., Sitz T., Abdalla M.A., Mühling K.H., Rohn S. (2023). LC-ESI-MS/MS Analysis of Sulfolipids and Galactolipids in Green and Red Lettuce (*Lactuca sativa* L.) as Influenced by Sulfur Nutrition. Int. J. Mol. Sci..

[124] Hellgren L.I. (2001). Occurrence of bioactive sphingolipids in meat and fish products. Eur. J. Lipid Sci. Technol..

[125] Kim S.B., Bisson J., Friesen J.B., Pauli G.F., Simmler C. (2020). Selective Chlorophyll Removal Method to “Degreen” Botanical Extracts. J. Nat. Prod..

[126] Van Echten-Deckert G., Merrill A.H., Hannun Y.A. (2000). Sphingolipid Extraction and Analysis by Thin-Layer Chromatography. Methods in Enzymology.

[127] Schnaar R.L., Sandhoff R., Tiemeyer M., Kinoshita T. (2022). Glycosphingolipids. Essentials of Glycobiology.

[128] Christie W.W., Han X., Christie W.W., Han X. (2012). Chromatographic analysis of sphingolipids. Lipid Analysis.

[129] Noda A., Kato M., Miyazaki S., Kyogashima M. (2018). Separation of glycosphingolipids with titanium dioxide. Glycoconj. J..

[130] Nagasawa H., Miyazaki S., Kyogashima M. (2022). Simple separation of glycosphingolipids in the lower phase of a Folch’s partition from crude lipid fractions using zirconium dioxide. Glycoconj. J..

[131] Sørensen L.K. (2006). A liquid chromatography/tandem mass spectrometric approach for the determination of gangliosides GD3 and GM3 in bovine milk and infant formulae. Rapid Commun. Mass Spectrom..

[132] Smith D.F., Prieto P.A. (1993). Special Considerations for Glycolipids and Their Purification. Curr. Protoc. Mol. Biol..

[133] Ledeen R.W., Yu R.K., Eng L.F. (1973). Gangliosides of human myelin: Sialosylgalactosylceramide (g7) as a major component. J. Neurochem..

[134] Hunter G.D., Wiegant V.M., Dunn A.J. (1981). Interspecies Comparison of Brain Ganglioside Patterns Studied by Two-Dimensional Thin-Layer Chromatography. J. Neurochem..

[135] Ikeda K., Shimizu T., Taguchi R. (2008). Targeted analysis of ganglioside and sulfatide molecular species by LC/ESI-MS/MS with theoretically expanded multiple reaction monitoring. J. Lipid Res..

[136] Schnaar R.L., Needham L.K. (1994). Thin-layer chromatography of glycosphingolipids. Methods in Enzymology.

[137] Smyth T.J.P., Rudden M., Tsaousi K., Marchant R., Banat I.M., McGenity T.J., Timmis K.N., Nogales B. (2016). Protocols for the Detection and Chemical Characterisation of Microbial Glycolipids. Hydrocarbon and Lipid Microbiology Protocols: Biochemical Methods.

[138] Smyth T.J.P., Perfumo A., Marchant R., Banat I.M., Timmis K.N. (2010). Isolation and Analysis of Low Molecular Weight Microbial Glycolipids. Handbook of Hydrocarbon and Lipid Microbiology.

[139] Koch A.K., Käppeli O., Fiechter A., Reiser J. (1991). Hydrocarbon assimilation and biosurfactant production in *Pseudomonas aeruginosa* mutants. J. Bacteriol..

[140] Aveiro S.S., Melo T., Figueiredo A., Domingues P., Pereira H., Maia I.B., Silva J., Domingues M.R., Nunes C., Moreira A.S.P. (2020). The Polar Lipidome of Cultured Emiliania huxleyi: A Source of Bioactive Lipids with Relevance for Biotechnological Applications. Biomolecules.

[141] Bell B.M., Daniels D.G.H., Fearn T., Stewart B.A. (1987). Lipid compositions, baking qualities and other characteristics of wheat varieties grown in the U.K. J. Cereal Sci..

[142] Fuchs B., Süß R., Teuber K., Eibisch M., Schiller J. (2011). Lipid analysis by thin-layer chromatography—A review of the current state. J. Chromatogr. A.

[143] Palumbo G., Zullo F. (1987). The use of iodine staining for the quantitative analysis of lipids separated by thin layer chromatography. Lipids.

[144] Irie T., Watarai S., Kushi Y., Kasama T., Kodama H. (2004). Analysis of gangliosides from carp intestinal mucosa. Fish Shellfish Immunol..

[145] Müthing J., Distler U. (2010). Advances on the compositional analysis of glycosphingolipids combining thin-layer chromatography with mass spectrometry. Mass Spectrom. Rev..

[146] Yoshio H., Keiko K., Hideyoshi H., Yasuo S., Makoto M., Mamoru S., Tomoya O. (1986). Sensitive enzyme-immunostaining and densitometric determination of ganglio-series gangliosides on thin-layer plate: Pmol detection of gangliosides in cerebrospinal fluid. Biochim. Biophys. Acta BBA-Lipids Lipid Metab..

[147] Viljetić B., Labak I., Blažetić S., Stambuk A., Heffer M. (2012). Distribution of mono-, di- and trisialo gangliosides in the brain of *Actinopterygian fishes*. Biochim. Biophys. Acta.

[148] Kates M., Kates M. (1990). Glycolipids of Higher Plants, Algae, Yeasts, and Fungi. Glycolipids, Phosphoglycolipids, and Sulfoglycolipids.

[149] Nakamura K., Suzuki Y., Goto-Inoue N., Yoshida-Noro C., Suzuki A. (2006). Structural Characterization of Neutral Glycosphingolipids by Thin-Layer Chromatography Coupled to Matrix-Assisted Laser Desorption/Ionization Quadrupole Ion Trap Time-of-Flight MS/MS. Anal. Chem..

[150] Müthing J. (1996). High-resolution thin-layer chromatography of gangliosides. J. Chromatogr. A.

[151] Harth S., Dreyfus H., Urban P.F., Mandel P. (1978). Direct thin-layer chromatography of gangliosides of a total lipid extract. Anal. Biochem..

[152] Eichenberger W., Araki S., Müller D.G. (1993). Betaine lipids and phospholipids in brown algae. Phytochemistry.

[153] Dembitsky V.M., Pechenkina-Shubina E.E., Rozentsvet O.A. (1991). Glycolipids and fatty acids of some seaweeds and marine grasses from the black sea. Phytochemistry.

[154] Neskovic N., Sarlieve L., Nussbaum J.-L., Kostic D., Mandel P. (1972). Quantitative thin-layer chromatography of glycolipids in animal tissues. Clin. Chim. Acta.

[155] Olsen R.E., Henderson R.J. (1989). The rapid analysis of neutral and polar marine lipids using double-development HPTLC and scanning densitometry. J. Exp. Mar. Biol. Ecol..

[156] Avrova N.F., Ghidoni R., Karpova O.B., Nalivayeva N.N., Malesci A., Tettamanti G. (1986). Systematic position of fish species and ganglioside composition and content. Comp. Biochem. Physiol. Part B Comp. Biochem..

[157] Scandroglio F., Loberto N., Valsecchi M., Chigorno V., Prinetti A., Sonnino S. (2009). Thin layer chromatography of gangliosides. Glycoconj. J..

[158] Becker K., Wöhrmann A.P.A., Rahmann H. (1995). Brain gangliosides and cold-adaptation in high-antarctic fish. Biochem. Syst. Ecol..

[159] Mauri L., Sonnino S. (2023). Alkali-labile gangliosides. Glycoconj. J..

[160] Poole C.F., Poole C.F. (2023). High-performance precoated stationary phases. Instrumental Thin-Layer Chromatography.

[161] Fine J.B., Sprecher H. (1982). Unidimensional thin-layer chromatography of phospholipids on boric acid-impregnated plates. J. Lipid Res..

[162] Deranieh R.M., Joshi A.S., Greenberg M.L., Rapaport D., Herrmann J.M. (2013). Thin-Layer Chromatography of Phospholipids. Membrane Biogenesis: Methods and Protocols.

[163] Pinault M., Guimaraes C., Dumas J.-F., Servais S., Chevalier S., Besson P., Goupille C. (2020). A 1D High Performance Thin Layer Chromatography Method Validated to Quantify Phospholipids Including Cardiolipin and Monolysocardiolipin from Biological Samples. Eur. J. Lipid Sci. Technol..

[164] Sobańska A.W. (2020). Impregnated silica-based layers in thin layer chromatography. J. Liq. Chromatogr. Relat. Technol..

[165] Bodennec J., Pelled D., Futerman A.H. (2003). Aminopropyl solid phase extraction and 2 D TLC of neutral glycosphingolipids and neutral lysoglycosphingolipids. J. Lipid Res..

[166] Bitman J., Wood D.L. (1981). Quantitative Densitometry in Situ of Lipids Separated by thin Layer Chromatography. J. Liq. Chromatogr..

[167] Macala L., Yu R., Ando S. (1983). Analysis of brain lipids by high performance thin-layer chromatography and densitometry. J. Lipid Res..

[168] Meullemiestre A., Breil C., Abert-Vian M., Chemat F., Meullemiestre A., Breil C., Abert-Vian M., Chemat F. (2015). Analytical Methodology for Lipid Extraction and Quantification from Oleaginous Microorganisms. Modern Techniques and Solvents for the Extraction of Microbial Oils.

[169] Cebolla V.L., Jarne C., Vela J., Garriga R., Membrado L., Galbán J. (2021). Scanning densitometry and mass spectrometry for HPTLC analysis of lipids: The last 10 years. J. Liq. Chromatogr. Relat. Technol..

[170] Watanabe K., Mizuta M. (1995). Fluorometric detection of glycosphingolipids on thin-layer chromatographic plates. J. Lipid Res..

[171] Christie W.W., Han X., Christie W.W., Han X. (2012). Chromatographic analysis of lipids: General principles. Lipid Analysis.

[172] Cebolla V.L., Mateos E., Garriga R., Jarne C., Membrado L., Cossío F.P., Gálvez E.M., Matt M., Delgado-Camón A. (2012). Changes in Fluorescent Emission Due to Non-covalent Interactions as a General Detection Procedure for Thin-Layer Chromatography. ChemPhysChem.

[173] Suzuki A., Miyazaki M., Matsuda J., Yoneshige A. (2011). High-performance thin-layer chromatography/mass spectrometry for the analysis of neutral glycosphingolipids. Biochim. Biophys. Acta BBA-Mol. Cell Biol. Lipids.

[174] Domínguez A., Jarne C., Cebolla V.L., Galbán J., Savirón M., Orduna J., Membrado L., Lapieza M.-P., Romero E., Sanz Vicente I. (2015). A Hyphenated Technique based on High-Performance Thin Layer Chromatography for Determining Neutral Sphingolipids: A Proof of Concept. Chromatography.

[175] Sarbu M., Zamfir A.D., El Rassi Z. (2021). Modern techniques for separation, mass spectrometric detection, and characterization of glycolipids. Carbohydrate Analysis by Modern Liquid Phase Separation Techniques.

[176] Niimura Y., Tomori M., Tadano-Aritomi K., Toida T., Ishizuka I. (1999). The major acidic glycolipids from the kidney of the Pacific salmon (*Oncorhynchus keta*): Characterization of a novel ganglioside, fucosyl-N-acetylgalactosaminyl-GM1. J. Biochem..

[177] Tadano-Aritomi K., Ishizuka I. (1983). Determination of peracetylated sulfoglycolipids using the azure A method. J. Lipid Res..

[178] Bethke U., Müthing J., Schauder B., Conradt P., Mühlradt P.F. (1986). An improved semi-quantitative enzyme immunostaining procedure for glycosphingolipid antigens on high performance thin layer chromatograms. J. Immunol. Methods.

[179] Anyakudo F., Adams E., Van Schepdael A. (2020). Thin-Layer Chromatography–Flame Ionization Detection. Chromatographia.

[180] Nahar A., Baker A.L., Nichols D.S., Bowman J.P., Britz M.L. (2020). Application of Thin-Layer Chromatography-Flame Ionization Detection (TLC-FID) to Total Lipid Quantitation in Mycolic-Acid Synthesizing Rhodococcus and Williamsia Species. Int. J. Mol. Sci..

[181] Sinanoglou V.J., Strati I.F., Bratakos S.M., Proestos C., Zoumpoulakis P., Miniadis-Meimaroglou S. (2013). On the combined application of Iatroscan TLC-FID and GC-FID to identify total, neutral, and polar lipids and their fatty acids extracted from foods. Int. Sch. Res. Not..

[182] Parrish C.C. (1987). Separation of Aquatic Lipid Classes by Chromarod Thin-Layer Chromatography with Measurement by latroscan Flame Ionization Detection. Can. J. Fish. Aquat. Sci..

[183] Parrish C.C., Ackman R.G. (1983). Chromarod separations for the analysis of marine lipid classes by iatroscan chromatography-flame ionization detection. J. Chromatogr. A.

[184] Habeebullah S.F.K., Alagarsamy S., Haddad S.A., Yamani F.A. (2023). Composition, In vitro Antioxidant and Angiotensin-Converting Enzyme Inhibitory Effects of Lipids Isolated from Fifteen Species of Seaweeds. Food Chem. Adv..

[185] Park H., Zhou Y., Costello C.E. (2014). Direct analysis of sialylated or sulfated glycosphingolipids and other polar and neutral lipids using TLC-MS interfaces. J. Lipid Res..

[186] Engel K.M., Prabutzki P., Leopold J., Nimptsch A., Lemmnitzer K., Vos D.R.N., Hopf C., Schiller J. (2022). A new update of MALDI-TOF mass spectrometry in lipid research. Prog. Lipid Res..

[187] Paglia G., Ifa D.R., Wu C., Corso G., Cooks R.G. (2010). Desorption Electrospray Ionization Mass Spectrometry Analysis of Lipids after Two-Dimensional High-Performance Thin-Layer Chromatography Partial Separation. Anal. Chem..

[188] Eberlin L.S., Ferreira C.R., Dill A.L., Ifa D.R., Cooks R.G. (2011). Desorption electrospray ionization mass spectrometry for lipid characterization and biological tissue imaging. Biochim. Biophys. Acta.

[189] Miao Z., Chen H. (2009). Direct analysis of liquid samples by desorption electrospray ionization-mass spectrometry (DESI-MS). J. Am. Soc. Mass Spectrom..

[190] Himmelsbach M., Varesio E., Hopfgartner G. (2014). Liquid extraction surface analysis (LESA) of hydrophobic TLC plates coupled to chip-based nanoelectrospray high-resolution mass spectrometry. Chimia.

[191] Fuchs B. (2012). Analysis of phospolipids and glycolipids by thin-layer chromatography–matrix-assisted laser desorption and ionization mass spectrometry. J. Chromatogr. A.

[192] Leopold J., Prabutzki P., Engel K.M., Schiller J. (2023). A Five-Year Update on Matrix Compounds for MALDI-MS Analysis of Lipids. Biomolecules.

[193] Jaskolla T.W., Karas M. (2011). Compelling Evidence for Lucky Survivor and Gas Phase Protonation: The Unified MALDI Analyte Protonation Mechanism. J. Am. Soc. Mass Spectrom..

[194] Fuchs B., Schiller J., Süß R., Nimptsch A., Schürenberg M., Suckau D. (2009). Capabilities and disadvantages of combined matrix-assisted laser-desorption/ionization time-of-flight mass spectrometry (MALDI-TOF MS) and high-performance thin-layer chromatography (HPTLC): Analysis of egg yolk lipids. JPC-J. Planar Chromatogr.-Mod. TLC.

[195] McMillen J.C., Fincher J.A., Klein D.R., Spraggins J.M., Caprioli R.M. (2020). Effect of MALDI matrices on lipid analyses of biological tissues using MALDI-2 postionization mass spectrometry. J. Mass Spectrom..

[196] Juhasz P., Costello C.E. (1992). Matrix-assisted laser desorption ionization time-of-flight mass spectrometry of underivatized and permethylated gangliosides. J. Am. Soc. Mass Spectrom..

[197] Kim S.W., Kwon S., Kim Y.K. (2021). Graphene Oxide Derivatives and Their Nanohybrid Structures for Laser Desorption/Ionization Time-of-Flight Mass Spectrometry Analysis of Small Molecules. Nanomaterials.

[198] Lu M., Yang X., Yang Y., Qin P., Wu X., Cai Z. (2017). Nanomaterials as Assisted Matrix of Laser Desorption/Ionization Time-of-Flight Mass Spectrometry for the Analysis of Small Molecules. Nanomaterials.

[199] Cha S., Yeung E.S. (2007). Colloidal Graphite-Assisted Laser Desorption/Ionization Mass Spectrometry and MSn of Small Molecules. 1. Imaging of Cerebrosides Directly from Rat Brain Tissue. Anal. Chem..

[200] Hua P.-Y., Manikandan M., Abdelhamid H.N., Wu H.-F. (2014). Graphene nanoflakes as an efficient ionizing matrix for MALDI-MS based lipidomics of cancer cells and cancer stem cells. J. Mater. Chem. B.

[201] Wang Z., Cai Y., Wang Y., Zhou X., Zhang Y., Lu H. (2017). Improved MALDI imaging MS analysis of phospholipids using graphene oxide as new matrix. Sci. Rep..

[202] Kertesz V., Van Berkel G.J. (2010). Fully automated liquid extraction-based surface sampling and ionization using a chip-based robotic nanoelectrospray platform. J. Mass Spectrom..

[203] Dill A.L., Ifa D.R., Manicke N.E., Ouyang Z., Cooks R.G. (2009). Mass spectrometric imaging of lipids using desorption electrospray ionization. J. Chromatogr. B Anal. Technol. Biomed. Life Sci..

[204] Takáts Z., Wiseman J.M., Gologan B., Cooks R.G. (2004). Mass spectrometry sampling under ambient conditions with desorption electrospray ionization. Science.

[205] Ifa D.R., Wu C., Ouyang Z., Cooks R.G. (2010). Desorption electrospray ionization and other ambient ionization methods: Current progress and preview. Analyst.

[206] Das S., Bhatia R. (2022). Liquid extraction surface analysis-mass spectrometry: An advanced and environment-friendly analytical tool in modern analysis. J. Sep. Sci..

[207] Brown S.H., Huxtable L.H., Willcox M.D., Blanksby S.J., Mitchell T.W. (2013). Automated surface sampling of lipids from worn contact lenses coupled with tandem mass spectrometry. Analyst.

[208] Jarne C., Membrado L., Saviron M., Vela J., Orduna J., Garriga R., Galban J., Cebolla V.L. (2021). Globotriaosylceramide-related biomarkers of fabry disease identified in plasma by high-performance thin-layer chromatography—Densitometry—Mass spectrometry. J. Chromatogr. A.

[209] Leopold J., Popkova Y., Engel K.M., Schiller J. (2018). Recent Developments of Useful MALDI Matrices for the Mass Spectrometric Characterization of Lipids. Biomolecules.

[210] Fraser A.J., Tocher D.R., Sargent J.R. (1985). Thin-layer chromatography—Flame ionization detection and the quantitation of marine neutral lipids and phospholipids. J. Exp. Mar. Biol. Ecol..

[211] Ackman R.G. (1981). Flame ionization detection applied to thin-layer chromatography on coated quartz rods. Methods Enzymol..

[212] Buszewski B., Noga S. (2012). Hydrophilic interaction liquid chromatography (HILIC)—A powerful separation technique. Anal. Bioanal. Chem..

[213] Alpert A.J. (1990). Hydrophilic-interaction chromatography for the separation of peptides, nucleic acids and other polar compounds. J. Chromatogr. A.

[214] Chauve B., Guillarme D., Cléon P., Veuthey J.L. (2010). Evaluation of various HILIC materials for the fast separation of polar compounds. J. Sep. Sci..

[215] Schwalbe-Herrmann M., Willmann J., Leibfritz D. (2010). Separation of phospholipid classes by hydrophilic interaction chromatography detected by electrospray ionization mass spectrometry. J. Chromatogr. A.

[216] Jandera P., Janás P. (2017). Recent advances in stationary phases and understanding of retention in hydrophilic interaction chromatography. A review. Anal. Chim. Acta.

[217] Qing G., Yan J., He X., Li X., Liang X. (2020). Recent advances in hydrophilic interaction liquid interaction chromatography materials for glycopeptide enrichment and glycan separation. Trends Anal. Chem..

[218] Jandera P. (2011). Stationary and mobile phases in hydrophilic interaction chromatography: A review. Anal. Chim. Acta.

[219] Oyler A.R., Armstrong B.L., Cha J.Y., Zhou M.X., Yang Q., Robinson R.I., Dunphy R., Burinsky D.J. (1996). Hydrophilic interaction chromatography on amino-silica phases complements reversed-phase high-performance liquid chromatography and capillary electrophoresis for peptide analysis. J. Chromatogr. A.

[220] Bartosova Z., Gonzalez S.V., Voigt A., Bruheim P. (2021). High Throughput Semiquantitative UHPSFC–MS/MS Lipid Profiling and Lipid Class Determination. J. Chromatogr. Sci..

[221] Koelmel J.P., Cochran J.A., Ulmer C.Z., Levy A.J., Patterson R.E., Olsen B.C., Yost R.A., Bowden J.A., Garrett T.J. (2019). Software tool for internal standard based normalization of lipids, and effect of data-processing strategies on resulting values. BMC Bioinform..

[222] Li Z., Wang X., Deng X., Song J., Yang T., Liao Y., Gong G., Huang L., Lu Y., Wang Z. (2023). High-sensitivity qualitative and quantitative analysis of human, bovine and goat milk glycosphingolipids using HILIC-MS/MS with internal standards. Carbohydr. Polym..

[223] Wang M., Wang C., Han X. (2017). Selection of internal standards for accurate quantification of complex lipid species in biological extracts by electrospray ionization mass spectrometry—What, how and why?. Mass Spectrom. Rev..

[224] Lange M., Fedorova M. (2020). Evaluation of lipid quantification accuracy using HILIC and RPLC MS on the example of NIST^®^ SRM^®^ 1950 metabolites in human plasma. Anal. Bioanal. Chem..

[225] Fong B., Norris C., Lowe E., McJarrow P. (2009). Liquid chromatography-high-resolution mass spectrometry for quantitative analysis of gangliosides. Lipids.

[226] Inoue S., Kitajima K. (2006). KDN (deaminated neuraminic acid): Dreamful past and exciting future of the newest member of the sialic acid family. Glycoconj. J..

[227] Schnaar R.L., Gerardy-Schahn R., Hildebrandt H. (2014). Sialic acids in the brain: Gangliosides and polysialic acid in nervous system development, stability, disease, and regeneration. Physiol. Rev..

[228] Li H., Song Y., Zhang H., Wang X., Cong P., Xu J., Xue C. (2020). Comparative lipid profile of four edible shellfishes by UPLC-Triple TOF-MS/MS. Food Chem..

[229] Hu X., Cong P., Song Y., Wang X., Zhang H., Meng N., Fan X., Xu J., Xue C. (2023). Comprehensive Lipid Profile of Eight Echinoderm Species by RPLC–Triple TOF-MS/MS. J. Agric. Food Chem..

[230] Wang X., Zhang H., Song Y., Cong P., Li Z., Xu J., Xue C. (2019). Comparative Lipid Profile Analysis of Four Fish Species by Ultraperformance Liquid Chromatography Coupled with Quadrupole Time-of-Flight Mass Spectrometry. J. Agric. Food Chem..

[231] Windarsih A., Irnawati, Suratno, Warmiko H.D., Alam L.P.M., Utami I.D., Rohman A., Indrianingsih A.W. (2024). Lipidomics Analysis of Different Marine Fish Oils Using Untargeted Liquid Chromatography–Orbitrap High-Resolution Mass Spectrometry and Chemometrics. Chromatographia.

[232] da Costa E., Domingues P., Melo T., Coelho E., Pereira R., Calado R., Abreu M.H., Domingues M.R. (2019). Lipidomic Signatures Reveal Seasonal Shifts on the Relative Abundance of High-Valued Lipids from the Brown Algae *Fucus vesiculosus*. Mar. Drugs.

[233] Moreira A.S.P., da Costa E., Melo T., Sulpice R., Cardoso S.M., Pitarma B., Pereira R., Abreu M.H., Domingues P., Calado R. (2020). Seasonal plasticity of the polar lipidome of Ulva rigida cultivated in a sustainable integrated multi-trophic aquaculture. Algal Res..

[234] Popendorf K.J., Fredricks H.F., Van Mooy B.A. (2013). Molecular ion-independent quantification of polar glycerolipid classes in marine plankton using triple quadrupole MS. Lipids.

[235] Xu J., Duan J., Xue C., Feng T., Dong P., Sugawara T., Hirata T. (2011). Analysis and comparison of glucocerebroside species from three edible sea cucumbers using liquid chromatography-ion trap-time-of-flight mass spectrometry. J. Agric. Food Chem..

[236] Cong P.X., Gao R.C., Xue C.H., Li Z.J., Zhang H.W., Khan M.N., Xue Y., Sugawara T., Xu J. (2015). Molecular species analysis of monosialogangliosides from sea urchin Strongylocentrotus nudus by RPLC-ESI-MS/MS. Food Chem..

[237] Andersen R.J., Taglialatela-Scafati O. (2005). Avrainvilloside, a 6-Deoxy-6-aminoglucoglycerolipid from the Green Alga Avrainvillea nigricans. J. Nat. Prod..

[238] Zahran E.M., Sayed A.M., Abdelwahab M.F., Albohy A., Abdulrazik B.S., Ibrahim A.M., Bringmann G., Abdelmohsen U.R. (2021). Identifying the specific-targeted marine cerebrosides against SARS-CoV-2: An integrated computational approach. RSC Adv..

[239] Cífková E., Hájek R., Lísa M., Holčapek M. (2016). Hydrophilic interaction liquid chromatography-mass spectrometry of (lyso)phosphatidic acids, (lyso)phosphatidylserines and other lipid classes. J. Chromatogr. A.

[240] Shaner R.L., Allegood J.C., Park H., Wang E., Kelly S., Haynes C.A., Sullards M.C., Merrill A.H. (2009). Quantitative analysis of sphingolipids for lipidomics using triple quadrupole and quadrupole linear ion trap mass spectrometers[S]. J. Lipid Res..

[241] Brignol N., Chang K., Hamler R., Schilling A.E., Khanna R., Lockhart D.J., Clark S.W., Benjamin E.R. (2012). Glucosylceramide Quantitation in Normal and Glucocerebrosidase-Deficient Mouse Brain and Human Cell Lines. Mol. Genet. Metab..

[242] Zhu C., Dane A., Spijksma G., Wang M., van der Greef J., Luo G., Hankemeier T., Vreeken R.J. (2012). An efficient hydrophilic interaction liquid chromatography separation of 7 phospholipid classes based on a diol column. J. Chromatogr. A.

[243] Wang X., Li W., Rasmussen H.T. (2005). Orthogonal method development using hydrophilic interaction chromatography and reversed-phase high-performance liquid chromatography for the determination of pharmaceuticals and impurities. J. Chromatogr. A.

[244] Santalova E.A., Denisenko V.A., Dmitrenok P.S. (2015). Structural Analysis of the Minor Cerebrosides from a Glass Sponge *Aulosaccus* sp.. Lipids.

[245] Tsuji K., Mitsutake S., Ishikawa J., Takagi Y., Akiyama M., Shimizu H., Tomiyama T., Igarashi Y. (2006). Dietary glucosylceramide improves skin barrier function in hairless mice. J. Dermatol. Sci..

[246] Miyanishi K., Shiono N., Shirai H., Dombo M., Kimata H. (2005). Reduction of transepidermal water loss by oral intake of glucosylceramides in patients with atopic eczema. Allergy.

[247] Guillou S., Ghabri S., Jannot C., Gaillard E., Lamour I., Boisnic S. (2011). The moisturizing effect of a wheat extract food supplement on women’s skin: A randomized, double-blind placebo-controlled trial. Int. J. Cosmet. Sci..

[248] Fukunaga S., Wada S., Sato T., Hamaguchi M., Aoi W., Higashi A. (2018). Effect of Torula Yeast (*Candida utilis*)-Derived Glucosylceramide on Skin Dryness and Other Skin Conditions in Winter. J. Nutr. Sci. Vitaminol..

[249] Sugawara T. (2022). Sphingolipids as Functional Food Components: Benefits in Skin Improvement and Disease Prevention. J. Agric. Food Chem..

[250] Zábranská M., Vrkoslav V., Sobotníková J., Cvačka J. (2012). Analysis of plant galactolipids by reversed-phase high-performance liquid chromatography/mass spectrometry with accurate mass measurement. Chem. Phys. Lipids.

[251] Ibrahim A., Schütz A.-L., Galano J.-M., Herrfurth C., Feussner K., Durand T., Brodhun F., Feussner I. (2011). The Alphabet of Galactolipids in Arabidopsis thaliana. Front. Plant Sci..

[252] Napolitano A., Carbone V., Saggese P., Takagaki K., Pizza C. (2007). Novel Galactolipids from the Leaves of *Ipomoea batatas* L.: Characterization by Liquid Chromatography Coupled with Electrospray Ionization–Quadrupole Time-of-Flight Tandem Mass Spectrometry. J. Agric. Food Chem..

[253] Muggli T., Bühr C., Schürch S. (2022). Challenges in the Analysis of Gangliosides by LC-MS. Chimia.

[254] Gobburi A.L.P., Kipruto E.W., Inman D.M., Anderson D.J. (2021). A new LC-MS/MS technique for separation of gangliosides using a phenyl-hexyl column: Systematic separation according to sialic acid class and ceramide subclass. J. Liq. Chromatogr. Relat. Technol..

[255] Barrientos R.C., Zhang Q. (2018). Isobaric Labeling of Intact Gangliosides toward Multiplexed LC–MS/MS-Based Quantitative Analysis. Anal. Chem..

[256] Lee H., German J.B., Kjelden R., Lebrilla C.B., Barile D. (2013). Quantitative Analysis of Gangliosides in Bovine Milk and Colostrum-Based Dairy Products by Ultrahigh Performance Liquid Chromatography-Tandem Mass Spectrometry. J. Agric. Food Chem..

[257] Gordillo R. (2021). Supercritical fluid chromatography hyphenated to mass spectrometry for metabolomics applications. J. Sep. Sci..

[258] Chen L., Dean B., Liang X. (2021). A technical overview of supercritical fluid chromatography-mass spectrometry (SFC-MS) and its recent applications in pharmaceutical research and development. Drug Discov. Today Technol..

[259] Si-Hung L., Bamba T. (2022). Current state and future perspectives of supercritical fluid chromatography. Trends Anal. Chem..

[260] Yang Y., Liang Y., Yang J., Ye F., Zhou T., Gongke L. (2019). Advances of supercritical fluid chromatography in lipid profiling. J. Pharm. Anal..

[261] Donato P., Inferrera V., Sciarrone D., Mondello L. (2017). Supercritical fluid chromatography for lipid analysis in foodstuffs. J. Sep. Sci..

[262] Wolrab D., Chocholoušková M., Jirásko R., Peterka O., Holčapek M. (2020). Validation of lipidomic analysis of human plasma and serum by supercritical fluid chromatography–mass spectrometry and hydrophilic interaction liquid chromatography–mass spectrometry. Anal. Bioanal. Chem..

[263] Taguchi K., Fukusaki E., Bamba T. (2014). Simultaneous analysis for water- and fat-soluble vitamins by a novel single chromatography technique unifying supercritical fluid chromatography and liquid chromatography. J. Chromatogr. A.

[264] Desfontaine V., Losacco G.L., Gagnebin Y., Pezzatti J., Farrell W.P., González-Ruiz V., Rudaz S., Veuthey J.-L., Guillarme D. (2018). Applicability of supercritical fluid chromatography—Mass spectrometry to metabolomics. I—Optimization of separation conditions for the simultaneous analysis of hydrophilic and lipophilic substances. J. Chromatogr. A.

[265] Losacco G.L., Bennett R., Ahmad I.A.H., Barrientos R.C., DaSilva J.O., Dong Y., Schuppe A.W., Wang Z., Aiken S., Mangion I. (2022). Dual-Gradient Unified Chromatography: A New Paradigm for Versatility in Simultaneous Multicomponent Analysis. Angew. Chem. Int. Ed..

[266] Bamba T., Shimonishi N., Matsubara A., Hirata K., Nakazawa Y., Kobayashi A., Fukusaki E. (2008). High throughput and exhaustive analysis of diverse lipids by using supercritical fluid chromatography-mass spectrometry for metabolomics. J. Biosci. Bioeng..

[267] Lísa M., Jiránková T. (2022). Highly repeatable and selective ultrahigh-performance supercritical fluid chromatography—Mass spectrometry interclass separation in lipidomic studies. Microchem. J..

[268] Uchikata T., Matsubara A., Nishiumi S., Yoshida M., Fukusaki E., Bamba T. (2012). Development of oxidized phosphatidylcholine isomer profiling method using supercritical fluid chromatography/tandem mass spectrometry. J. Chromatogr. A.

[269] Yamada T., Uchikata T., Sakamoto S., Yokoi Y., Nishiumi S., Yoshida M., Fukusaki E., Bamba T. (2013). Supercritical fluid chromatography/Orbitrap mass spectrometry based lipidomics platform coupled with automated lipid identification software for accurate lipid profiling. J. Chromatogr. A.

[270] Lísa M., Holčapek M. (2015). High-Throughput and Comprehensive Lipidomic Analysis Using Ultrahigh-Performance Supercritical Fluid Chromatography–Mass Spectrometry. Anal. Chem..

[271] Schwaiger M., Schoeny H., El Abiead Y., Hermann G., Rampler E., Koellensperger G. (2019). Merging metabolomics and lipidomics into one analytical run. Analyst.

[272] Ling Y.S., Liang H.J., Lin M.H., Tang C.H., Wu K.Y., Kuo M.L., Lin C.Y. (2014). Two-dimensional LC-MS/MS to enhance ceramide and phosphatidylcholine species profiling in mouse liver. Biomed. Chromatogr..

[273] Lísa M., Cífková E., Holčapek M. (2011). Lipidomic profiling of biological tissues using off-line two-dimensional high-performance liquid chromatography–mass spectrometry. J. Chromatogr. A.

[274] Nie H., Liu R., Yang Y., Bai Y., Guan Y., Qian D., Wang T., Liu H. (2010). Lipid profiling of rat peritoneal surface layers by online normal- and reversed-phase 2D LC QToF-MS[S]. J. Lipid Res..

[275] Bang D.Y., Moon M.H. (2013). On-line two-dimensional capillary strong anion exchange/reversed phase liquid chromatography–tandem mass spectrometry for comprehensive lipid analysis. J. Chromatogr. A.

[276] Narváez-Rivas M., Vu N., Chen G.Y., Zhang Q. (2017). Off-line mixed-mode liquid chromatography coupled with reversed phase high performance liquid chromatography-high resolution mass spectrometry to improve coverage in lipidomics analysis. Anal. Chim. Acta.

[277] François I., Sandra P. (2009). Comprehensive supercritical fluid chromatography×reversed phase liquid chromatography for the analysis of the fatty acids in fish oil. J. Chromatogr. A.

[278] Holčapek M., Ovčačíková M., Lísa M., Cífková E., Hájek T. (2015). Continuous comprehensive two-dimensional liquid chromatography-electrospray ionization mass spectrometry of complex lipidomic samples. Anal. Bioanal. Chem..

[279] Pham T.H., Zaeem M., Fillier T.A., Nadeem M., Vidal N.P., Manful C., Cheema S., Cheema M., Thomas R.H. (2019). Targeting Modified Lipids during Routine Lipidomics Analysis using HILIC and C30 Reverse Phase Liquid Chromatography coupled to Mass Spectrometry. Sci. Rep..

[280] Lu N., Wei D., Chen F., Yang S.-T. (2012). Lipidomic profiling and discovery of lipid biomarkers in snow alga Chlamydomonas nivalis under salt stress. Eur. J. Lipid Sci. Technol..

[281] Zhang Y.Y., Qin L., Liu Y.X., Zhou D.Y., Xu X.B., Du M., Zhu B.W., Thornton M. (2018). Evaluation of lipid profile in different tissues of Japanese abalone Haliotis discus hannai Ino with UPLC-ESI-Q-TOF-MS-based lipidomic study. Food Chem..

[282] Zhu S., Ye M., Xu J., Guo C., Zheng H., Hu J., Chen J., Wang Y., Xu S., Yan X. (2015). Lipid Profile in Different Parts of Edible Jellyfish Rhopilema esculentum. J. Agric. Food Chem..

[283] de Souza L.M., Iacomini M., Gorin P.A.J., Sari R.S., Haddad M.A., Sassaki G.L. (2007). Glyco- and sphingophosphonolipids from the medusa Phyllorhiza punctata: NMR and ESI-MS/MS fingerprints. Chem. Phys. Lipids.

[284] Lee H.G., Joo M., Park J.M., Kim M.A., Mok J., Cho S.H., Sohn Y.C., Lee H. (2022). Lipid Profiling of Pacific Abalone (*Haliotis discus hannai*) at Different Developmental Stages Using Ultrahigh Performance Liquid Chromatography-Tandem Mass Spectrometry. J. Anal. Methods Chem..

[285] Tsugawa H., Cajka T., Kind T., Ma Y., Higgins B., Ikeda K., Kanazawa M., VanderGheynst J., Fiehn O., Arita M. (2015). MS-DIAL: Data-independent MS/MS deconvolution for comprehensive metabolome analysis. Nat. Methods.

[286] Kind T., Liu K.-H., Lee D.Y., DeFelice B., Meissen J.K., Fiehn O. (2013). LipidBlast in silico tandem mass spectrometry database for lipid identification. Nat. Methods.

[287] Tsugawa H., Ikeda K., Takahashi M., Satoh A., Mori Y., Uchino H., Okahashi N., Yamada Y., Tada I., Bonini P. (2020). A lipidome atlas in MS-DIAL 4. Nat. Biotechnol..

[288] Horai H., Arita M., Kanaya S., Nihei Y., Ikeda T., Suwa K., Ojima Y., Tanaka K., Tanaka S., Aoshima K. (2010). MassBank: A public repository for sharing mass spectral data for life sciences. J. Mass Spectrom..

[289] Wang M., Carver J.J., Phelan V.V., Sanchez L.M., Garg N., Peng Y., Nguyen D.D., Watrous J., Kapono C.A., Luzzatto-Knaan T. (2016). Sharing and community curation of mass spectrometry data with Global Natural Products Social Molecular Networking. Nat. Biotechnol..

[290] Koelmel J.P., Kroeger N.M., Ulmer C.Z., Bowden J.A., Patterson R.E., Cochran J.A., Beecher C.W.W., Garrett T.J., Yost R.A. (2017). LipidMatch: An automated workflow for rule-based lipid identification using untargeted high-resolution tandem mass spectrometry data. BMC Bioinform..

[291] Koelmel J.P., Li X., Stow S.M., Sartain M.J., Murali A., Kemperman R., Tsugawa H., Takahashi M., Vasiliou V., Bowden J.A. (2020). Lipid Annotator: Towards Accurate Annotation in Non-Targeted Liquid Chromatography High-Resolution Tandem Mass Spectrometry (LC-HRMS/MS) Lipidomics Using A Rapid and User-Friendly Software. Metabolites.

[292] von Gerichten J., Saunders K., Bailey M.J., Gethings L.A., Onoja A., Geifman N., Spick M. (2024). Challenges in Lipidomics Biomarker Identification: Avoiding the Pitfalls and Improving Reproducibility. Metabolites.

[293] Köfeler H.C., Eichmann T.O., Ahrends R., Bowden J.A., Danne-Rasche N., Dennis E.A., Fedorova M., Griffiths W.J., Han X., Hartler J. (2021). Quality control requirements for the correct annotation of lipidomics data. Nat. Commun..

[294] Gonzalez-Riano C., León-Espinosa G., Regalado-Reyes M., García A., DeFelipe J., Barbas C. (2025). Advanced lipidomics using UHPLC-ESI-QTOF-MS/MS reveals novel lipids in hibernating syrian hamsters. J. Chromatogr. A.

[295] Xu H., Jiang T., Lin Y., Zhang L., Yang H., Huang X., Mao R., Yang Z., Zeng C., Zhao S. (2025). LipidIN: A comprehensive repository for flash platform-independent annotation and reverse lipidomics. Nat. Commun..

[296] Matsubara M., Ishihara M., Tiemeyer M., Aoki K., Ranzinger R. (2025). DANGO: An MS data annotation tool for glycolipidomics. BBA Adv..

[297] Weatherly D.B., Arpinar F.S., Porterfield M., Tiemeyer M., York W.S., Ranzinger R. (2019). GRITS Toolbox—A freely available software for processing, annotating and archiving glycomics mass spectrometry data. Glycobiology.

[298] Hoffmann N., Mayer G., Has C., Kopczynski D., Al Machot F., Schwudke D., Ahrends R., Marcus K., Eisenacher M., Turewicz M. (2022). A Current Encyclopedia of Bioinformatics Tools, Data Formats and Resources for Mass Spectrometry Lipidomics. Metabolites.

[299] Züllig T., Köfeler H.C. (2021). High Resolution Mass Spectrometry in Lipidomics. Mass Spectrom. Rev..

[300] Paglia G., Smith A.J., Astarita G. (2022). Ion mobility mass spectrometry in the omics era: Challenges and opportunities for metabolomics and lipidomics. Mass Spectrom. Rev..

[301] Harris R.A., Leaptrot K.L., May J.C., McLean J.A. (2019). New frontiers in lipidomics analyses using structurally selective ion mobility-mass spectrometry. Trends Anal. Chem..

[302] Paglia G., Angel P., Williams J.P., Richardson K., Olivos H.J., Thompson J.W., Menikarachchi L., Lai S., Walsh C., Moseley A. (2015). Ion mobility-derived collision cross section as an additional measure for lipid fingerprinting and identification. Anal. Chem..

[303] Camunas-Alberca S.M., Moran-Garrido M., Sáiz J., Gil-de-la-Fuente A., Barbas C., Gradillas A. (2023). Integrating the potential of ion mobility spectrometry-mass spectrometry in the separation and structural characterisation of lipid isomers. Front. Mol. Biosci..

[304] Naylor C.N., Nagy G. (2025). Recent advances in high-resolution traveling wave-based ion mobility separations coupled to mass spectrometry. Mass Spectrom. Rev..

[305] Wojcik R., Webb I.K., Deng L., Garimella S.V.B., Prost S.A., Ibrahim Y.M., Baker E.S., Smith R.D. (2017). Lipid and Glycolipid Isomer Analyses Using Ultra-High Resolution Ion Mobility Spectrometry Separations. Int. J. Mol. Sci..

[306] Wormwood Moser K.L., Van Aken G., DeBord D., Hatcher N.G., Maxon L., Sherman M., Yao L., Ekroos K. (2021). High-defined quantitative snapshots of the ganglioside lipidome using high resolution ion mobility SLIM assisted shotgun lipidomics. Anal. Chim. Acta.

[307] Naylor C.N., Nagy G. (2023). Permethylation and Metal Adduction: A Toolbox for the Improved Characterization of Glycolipids with Cyclic Ion Mobility Separations Coupled to Mass Spectrometry. Anal. Chem..

[308] Poad B.L.J., Zheng X., Mitchell T.W., Smith R.D., Baker E.S., Blanksby S.J. (2018). Online Ozonolysis Combined with Ion Mobility-Mass Spectrometry Provides a New Platform for Lipid Isomer Analyses. Anal. Chem..

